# Global well-posedness and interior regularity of 2D Navier–Stokes equations with stochastic boundary conditions

**DOI:** 10.1007/s00208-024-02812-0

**Published:** 2024-02-27

**Authors:** Antonio Agresti, Eliseo Luongo

**Affiliations:** 1 Institute of Science and Technology Austria (ISTA), Am Campus 1, 3400 Klosterneuburg, Austria; 2 Present Address: Delft Institute of Applied Mathematics, Delft University of Technology, P.O. Box 5031, 2600 GA Delft, The Netherlands; 3 Scuola Normale Superiore, Piazza dei Cavalieri, 7, 56126 Pisa, Italy

**Keywords:** 60H15, 76D03 (47A60, 35J25)

## Abstract

The paper is devoted to the analysis of the global well-posedness and the interior regularity of the 2D Navier–Stokes equations with inhomogeneous stochastic boundary conditions. The noise, white in time and coloured in space, can be interpreted as the physical law describing the driving mechanism on the atmosphere–ocean interface, i.e. as a balance of the shear stress of the ocean and the horizontal wind force.

## Introduction

Partial differential equations with boundary noise have been introduced by Da Prato and Zabczyck in the seminal paper [18]. They showed that, also in the one dimensional case, the solutions of the heat equation with white noise Dirichlet or Neumann Boundary conditions have low regularity compared to the case of noise diffused inside the domain. In particular, in the case of Dirichlet boundary conditions the solution is only a distribution. Some improvements in the analysis of the interior regularity of the solutions of these problems and some nonlinear variants have been obtained exploiting specific properties of the heat kernel and of suitable nonlinearities. For some results in this direction we refer to [4, 15, 23, 25, 31]. All these issues make the problem of treating non-linear partial differential equations with boundary noise coming from fluid dynamical models an, almost untouched, field of open problems.

Throughout the manuscript we fix a finite time horizon $T>0$. Let $a>0$, $\mathcal {O}=\mathbb {T}\times (0,a)$ and let $\mathbb {T}$ be the one dimensional torus. Finally, we denote by

1.1 $$\begin{aligned} \Gamma _b=\mathbb {T}\times \{0\}\quad \text { and }\quad \Gamma _u=\mathbb {T}\times \{a\}, \end{aligned}$$

the bottom and the upper part of the boundary of $\mathcal {O}$, respectively.

In this paper we are interested in the global well-posedness and the interior regularity of the 2D Navier–Stokes equations with boundary noise for the unknown velocity field $u(t,\omega ,x,z)=(u_1,u_2):\mathbb {R}_+\times \Omega \times \mathcal {O}\rightarrow \mathbb {R}^2$, formally written as

1.2 $$\begin{aligned} \left\{ \begin{aligned} \partial _{t}u+u\cdot \nabla u+\nabla P&=\Delta u, \qquad&\text { on }&(0,T)\times \mathcal {O}, \\ {\text {div}}u&=0,&\text { on }&(0,T)\times \mathcal {O}, \\ u&= 0,&\text { on }&(0,T)\times \Gamma _b,\\ \partial _{z}u_1&=h_b {\dot{W}}_{\mathcal {H}},&\text { on }&(0,T)\times \Gamma _u,\\ u_2&=0,&\text { on }&(0,T)\times \Gamma _u, \\ u( 0)&=u_0,&\text { on }&\mathcal {O}, \end{aligned} \right. \end{aligned}$$

where $\nabla u=(\partial _j u_i)_{i,j=1}^2$, $W_{\mathcal {H}}(t)$ is a $\mathcal {H}$-cylindrical Brownian motion and $h_b(t,x)$ is a sufficiently regular forcing term; we refer to Sect. 1.1 below for the the relevant assumptions and definitions. To the best of our knowledge this is the first instance of a *global* well-posedness result for a fluid dynamical system driven by stochastic white in time boundary conditions. We refer to [12, 13] for some homogenization results in the case of Navier–Stokes equations with dynamic boundary conditions driven by a stochastic forcing and to [14] for the local analysis of the three dimensional primitive equations with boundary noise. Finally, we refer to [21, 22] for some limit behaviors of the model (1.2) with $h_b {\dot{W}}_{\mathcal {H}}$ replaced by a highly oscillating and regular stationary random field.

Following the books by Pedlosky [40, 41] and Gill [30], the model (1.2) is a good idealization of the velocity of the fluid in the ocean. In this scenario, the domain $\mathcal {O}=\mathbb {T}\times (0,a)$ can be considered a vertical slice of the ocean with depth $a>0$ and we should interpret $u_1$ (resp. $u_2$) as the horizontal (resp. vertical) component of the velocity field *u*. Indeed even if, in principle, one should consider a free surface, instead of $\Gamma _u=\mathbb {T}\times \{a\}$, depending on the time, the approximation of such surface as independent of the time, although highly unrealistic, is justified by the fact that the behavior of the fluid around the surface is in general very turbulent. Hence, as emphasized in [24], only a modelization is tractable and meaningful. The stochastic boundary condition appearing in (1.2) is interpreted as the physical law describing the driving mechanism on the atmosphere-ocean interface, i.e. as a balance of the shear stress of the ocean and the horizontal wind force, see [38] for details.

### Main results

We begin by introducing some notation. Consider a complete filtered probability space $(\Omega ,\mathcal {F},(\mathcal {F}_t)_{t\ge 0},\textbf{P})$, a separable Hilbert space $\mathcal {H}$ and a cylindrical $\mathcal {F}-$Brownian motion $(W_{\mathcal {H}}(t))_{t\ge 0}$ on $\mathcal {H}$. We say that a process $\Phi$ is $\mathcal {F}$-progressive measurable if $\Phi |_{(0,t)\times \Omega }$ is $\mathcal {F}_t\times \mathcal {B}((0,t))$-measurable for all $t>0$, where $\mathcal {B}$ denotes the Borel $\sigma$-algebra. For the relevant notation on function spaces, we refer to Sect. 1.1.1.

#### Hypothesis 1.1

Let $q>2,\ p>2q,\ \alpha \in [0,\frac{1}{q}-\frac{2}{p})$ be such that there exists $\theta \in [0,\frac{1}{2})$ satisfying:

$$\begin{aligned} \theta +\frac{1}{4}-\frac{1}{p}\ge 0, \qquad -\frac{1}{q}-\alpha -2\theta +\frac{1}{2}> 0. \end{aligned}$$

Assume that $h_b:(0,T)\times \Omega \rightarrow W^{-\alpha ,q}(\Gamma _u;\mathcal {H})$ is a $\mathcal {F}$-progressively measurable process with $\textbf{P}-a.s.$ paths in $L^p(0,T;W^{-\alpha ,q}(\Gamma _u;\mathcal {H})).$

#### Remark 1.2

Hypothesis 1.1 is for instance satisfied if $q>2$, $p>2q>4$ and $\theta = \alpha =0$. Note that the case $q=2$ considered in [18] is not allowed in Hypothesis 1.1.

Following the idea of [17] we split the analysis of (1.2) in two parts. First we consider the stochastic linear problem with non-homogeneous boundary conditions

1.3 $$\begin{aligned} \left\{ \begin{aligned} \partial _{t}w+\nabla \rho&=\Delta w, \qquad \quad&\text { on }&(0,T)\times \mathcal {O},\\ {\text {div}}w&=0,&\text { on }&(0,T)\times \mathcal {O},\\ w&= 0,&\text { on }&(0,T)\times \Gamma _b,\\ \partial _{z}w_1&=h_b{\dot{W}}_{\mathcal {H}},&\text { on }&(0,T)\times \Gamma _u,\\ w_2&=0,&\text { on }&(0,T)\times \Gamma _u, \\ w (0)&=0,&\text { on }&\mathcal {O}, \end{aligned} \right. \end{aligned}$$

The solution to the above linear equation (1.3) can be treated in mild form as in [18, 19]. Secondly, denoting by $v=u-w$ we will consider the Navier–Stokes equations with random coefficients

1.4 $$\begin{aligned} \left\{ \begin{aligned} \partial _{t}v+(v+w)\cdot \nabla (v+w)+\nabla (P-\rho )&=\Delta v, \quad&\text { on }&(0,T)\times \mathcal {O},\\ {\text {div}}v&=0,&\text { on }&(0,T)\times \mathcal {O},\\ v&= 0,&\text { on }&(0,T)\times \Gamma _b,\\ \partial _{z}v_1&=0,&\text { on }&(0,T)\times \Gamma _u,\\ v_2&=0,&\text { on }&(0,T)\times \Gamma _u, \\ v(0)&=u_0,&\text { on }&\mathcal {O}. \end{aligned} \right. \end{aligned}$$

As discussed in [19, Chapter 13], if $h_b$, $u_0$, $W_{\mathcal {H}}(t)$ would be regular enough, then $u=v+w$ will be a classical solution of the Navier–Stokes equations with inhomogeneous boundary conditions (1.2).

To state our first result, we introduce some more notation. Here and below, we denote by *H* (resp. *V*, $\mathbb {L}^4$) the space $L^2(\mathcal {O};\mathbb {R}^2)$ (resp. $H^1(\mathcal {O};\mathbb {R}^2)$, $L^4(\mathcal {O};\mathbb {R}^2)$) of divergence free vector fields adapted to our framework, introduced rigorously in Sect. 2.1.

#### Definition 1.3

A process *u* with paths $\textbf{P}-a.s.$ in $C([0,T];H)\cap L^4(0,T;\mathbb {L}^4)$ and progressively measurable with respect to these topologies, is a pathwise weak solution of (1.2) if $u=v+w$, where *w* has paths in $C(0,T;H)\cap L^4(0,T;\mathbb {L}^4(\mathcal {O}))$, it is progressively measurable with respect to these topologies and is a mild solution of (1.3) while *v* has paths in $C(0,T;H)\cap L^2(0,T;V)$, it is progressively measurable with respect to these topologies and is a weak solution of (1.4).

The first main result of this paper reads as follows.

#### Theorem 1.4

(Global well-posedness) Let Hypothesis 1.1 be satisfied. Then for all $u_0\in H$ there exists a unique weak solution *u* to (1.2) in the sense of Definition 1.3.

According to Remark 1.2, the introduction of the non-Hilbertian setting is necessary in order to prove Theorem 1.4 above, at least with the tools introduced in this article.

#### Remark 1.5

(*Additional bulk forces*) Without additional difficulties we could also consider in Eq. (1.2) an additive noise diffused inside the domain of the form $h_d(t) \, \textrm{d}\widetilde{W}_{\mathcal {H}}(t)$, where $\widetilde{W}_{\mathcal {H}}$ is a cylindrical Brownian motion on $\mathcal {H}$ independent of $W_{\mathcal {H}}$ and $h_d:(0,T)\times \Omega \rightarrow \gamma (\mathcal {H}, X_{-\lambda ,A_q})$ is a progressively measurable process with paths $\textbf{P}-a.s.$ in $L^p\left( 0,T;\gamma \left( \mathcal {H},X_{-\lambda ,A_q}\right) \right)$, with $p>2,\ q\ge 2,\ \lambda \in [0,\frac{1}{2})$ such that $1-\frac{2}{p}-2\lambda >0$ and there exists $\theta \in [0,\frac{1}{2})$ satisfying

$$\begin{aligned} \theta \le \frac{3}{4}-\lambda -\frac{1}{q},\qquad \theta \ge \frac{1}{p}-\frac{1}{4}. \end{aligned}$$

The case $q=p=2$ and $\lambda =0$ is also allowed, see [20, Chapter 5]. Here $A_q$ and $\gamma$ stands for the Stokes operator on $L^q$ and the class of $\gamma$-radonifying operators, see Sect. 2.1 and [32, Chapter 9], respectively. Finally, $X_{-\lambda ,A_q}$ is the extrapolated space or order $\lambda$ w.r.t. $A_q$ as defined in (2.9). To see this, note that, under these assumptions, arguing as in Proposition 3.1 the solution $\widetilde{w}$ to

1.5 $$\begin{aligned} \left\{ \begin{aligned} \partial _t \widetilde{w}+\nabla \widetilde{\rho }&=\Delta \widetilde{w} +h_d \dot{\widetilde{W}}_{\mathcal {H}},\quad&\text { on }&(0,T)\times \mathcal {O},\\ {\text {div}}\widetilde{w}&=0,&\text { on }&(0,T)\times \mathcal {O}, \\ \widetilde{w}&= 0,&\text { on }&(0,T)\times \Gamma _b,\\ \partial _{z}\widetilde{w}_1&=0,&\text { on }&(0,T)\times \Gamma _u,\\ \widetilde{w}_2&=0,&\text { on }&(0,T)\times \Gamma _u, \\ \widetilde{w}(0)&=0,&\text { on }&\mathcal {O}, \end{aligned} \right. \end{aligned}$$

can be obtained as a stochastic convolution. In particular, the above assumptions on $h_d$ imply that $\widetilde{w}$ is a progressively measurable process with values in $C([0,T];H)\cap L^4(0,T;\mathbb {L}^4)$. Therefore this term adds no difficulties in order to analyze the well-posedness of Eq. (1.4). For this reason we prefer to not consider this classical source of randomness.

#### Remark 1.6

(*Comparison with the literature*)

1. Theorem 1.4 shares strong similarities with [11, Theorem 1.2], which addresses the well-posedness of certain 2D deterministic Navier–Stokes equations with non-homogeneous non-smooth Navier-type boundary conditions. However, it is important to note that our model focuses on a different phenomenon than the one studied in [11]. For this reason, contrary to us, they stress the regularity of the boundary condition of the normal trace of the velocity. From a mathematical viewpoint, the white noise appearing in Eq. (1.2) is rougher both in time and in space compared to the boundary conditions discussed in [11]. However, as discussed in [18], Neumann boundary conditions are more regular than Dirichlet boundary conditions and allow us to treat rougher inputs. Due to these differences, the two results have different ranges of applicability and do not cover each other. Moreover, the tools introduced here differ significantly from the techniques involved in [11].
2. As discussed in the introduction, the first result in the direction of the analysis of fluid dynamical models with stochastic boundary conditions have been proved in [14, Theorem 5.1], where the authors established local well-posedness of 3D primitive equations with boundary noise modeling wind forces. Both their strategy and ours are based on the splitting technique introduced in [17]. After showing suitable regularity properties of the stochastic convolution via stochastic maximal $L^p$-regularity techniques (cf. Proposition 3.1 and [14, Proposition 4.3]), a thorough analysis of certain nonlinear models is required. In contrast, we conduct this analysis within a suitable Hilbertian framework, enabling us to derive energy estimates essential for establishing the global well-posedness of (1.2) (cf. Theorem 3.3 and [14, Section 5.3]). The difference between the global well-posedness result which we are able to obtain and [14, Theorem 5.1] can be seen as consequence of the fact that the 2D Navier–Stokes equations are globally well-posed in the weak setting, while the same cannot be asserted for the primitive equations (cf. [33]). Therefore, in order to prove their local well-posedness result, the authors in [14] need to work with a notion of solution which mixes strong and weak regularity in the space variables. As a byproduct of this fact we are able to consider a noise rougher in space compared to them. Additionally, a minor distinction lies in the boundary conditions applied to the bottom part of the domain $\Gamma _b$. We introduce no-slip boundary conditions to accurately model the bottom of the ocean, a choice with theoretical underpinning in works such [21, 22, 30, 40, 41]. In contrast, [14] considered some form of homogeneous Neumann boundary conditions, a choice related to the functional analytic setup of the primitive equations (cf. [14, Remark 3.3]). Beyond the distinct justifications from a modeling perspective, our choice leads to differences in the analysis of the corresponding linear elliptic systems (cf. Section 2.2 and [14, Section 3.5]).

Secondly, we are interested in studying the interior regularity of the solution *u* provided by Theorem 1.4.

Our second main result reads as follows:

#### Theorem 1.7

(Interior regularity) Let Hypothesis 1.1 be satisfied. Let *u* be the unique weak solution of (1.2) provided by Theorem 1.4. Then for all $t_0\in (0,T)$ and $\mathcal {O}_0\subset \mathcal {O}$ such that $\textrm{dist}({\mathcal {O}_0}, \partial \mathcal {O})>0$,

$$\begin{aligned} u\in C([t_0,T]; C^{\infty }({\mathcal {O}_0};\mathbb {R}^2)) \quad \textbf{P}-a.s. \end{aligned}$$

According to [47] (see also [36, Section 13.1]), it seems not possible to gain high-order interior time-regularity for the Navier–Stokes problem. This fact is in contrast to the case of the heat equation with white noise boundary conditions, see [16]. The reason behind this is the presence of the unknown pressure *P* which, due to its non-local nature, provides a connection between the interior and the boundary regularity. Finally, let us mention that other techniques to bootstrap further interior space regularity (e.g. analyticity), such as the ‘parameter’ trick (see [7, 8] and [43, Subsection 9.4]), seem not to work due to the presence of the noise on $\Gamma _u$. Similarly to the proof of Theorem 1.4, we analyze the interior regularity of *u* combining the interior regularity of *w* and the interior regularity of *v*. The interior regularity of *w* is obtained introducing a proper weak formulation, see Definition 4.1 below. Instead the regularity of *v* is analyzed via a Serrin’s argument exploiting the aforementioned regularity of *w*.

The paper is organized as follows. In Sect. 2 we will introduce the functional setting in order to deal with problem (1.2). In particular, we will introduce the corresponding of the classical spaces and operator needed to deal with Navier–Stokes equations with no-slip boundary condition to this more involved set of boundary conditions. Indeed, the Stokes operator associated to our problem generates an analytic semigroup which admits an $H^{\infty }$-calculus of angle strictly less than $\frac{\pi }{2}$ also in the non-Hilbertian setting. This is crucial in order to apply the Stochastic maximal $L^p$-regularity results of [53], recalled in Sect. 2.4. The proof of Theorem 1.4 is the object of Sect. 3. In particular, in Sect. 3.1 we will consider the linear problem (1.3), while in Sect. 3.2 we will consider the nonlinear problem (1.4). The proof of Theorem 1.7 is the object of Sect. 4. In particular, in Sect. 4.1 we will study the interior regularity of the solution of the linear problem (1.3), while in Sect. 4.2 we will consider the nonlinear problem (1.4). We postpone some technical proofs related to the properties of the Stokes operator in the “Appendix A”.

#### Notation

Here we collect some notation which will be used throughout the paper. Further notation will be introduced where needed. By *C* we will denote several constants, perhaps changing value line by line. If we want to keep track of the dependence of *C* from some parameter $\xi$ we will use the symbol $C(\xi )$. Sometimes we will use the notation $a \lesssim b$ (resp. $a\lesssim _\xi b$), if it exists a constant such that $a \le C b$ (resp. $a\le C(\xi ) b$).

Fix $q\in (1,\infty )$. For an integer $k\ge 1$, $W^{k,q}$ denotes the usual Sobolev spaces. In the non-positive and non-integer case $s\in (-\infty ,\infty )\smallsetminus \mathbb {N}$, we let $W^{s,q}:=B^s_{q,q}$ where $B^{s}_{q,q}$ is the Besov space with smoothness *s*, and integrability *q* and microscopic integrability *q* (in particular $W^{0,q}\ne L^q$). Moreover, $H^{s,q}$ denotes the Bessel potential spaces. Both Besov and Bessel potential spaces can be defined by means of Littlewood-Paley decompositions and restrictions (see e.g. [46, 45, Section 6]) or using the interpolation methods starting with the standard Sobolev spaces $W^{k,q}$ (see e.g. [10, Chapter 6]). Finally, we set $\mathcal {A}^{s,q}(D;\mathbb {R}^d):=(\mathcal {A}^{s,q}(D))^d$ for an integer $d\ge 1$, a domain *D* and $\mathcal {A}\in \{W,H\}$.

Let $\mathcal {K}$ and *Y* be a Hilbert and a Banach space, respectively. We denote by $\gamma (\mathcal {K},Y)$ the set of $\gamma$-radonifying operators, see e.g. [32, Chapter 9] for basic definitions and properties. If *Y* is Hilbert, then $\gamma (\mathcal {K},Y)$ coincides with the class of Hilbert-Schmidt operator from $\mathcal {K}$ to *Y*. Below, we need the following Fubini-type result:

$$\begin{aligned} \mathcal {A}^{s,q}(D;\mathcal {K})=\gamma (\mathcal {K},\mathcal {A}^{s,q}(D))\ \ \text { for all }s\in \mathbb {R},\ q\in (1,\infty ), \ \mathcal {A}\in \{W,H\}. \end{aligned}$$

The above follows from [32, Theorem 9.3.6] and interpolation.

## Preliminaries

### The Stokes operator and its spectral properties

In this section we introduce the functional analytic setup in order to define all the object necessarily in the following. In order to improve the readability of the results we will just state the main results on the Stokes operator postponing the proofs to “Appendix A”.

Throughout this subsection we let $q\in (1,\infty )$. Recall that $\mathcal {O}=\mathbb {T}\times (0,a)$ where $a>0$. We begin by introducing the Helmholtz projection on $L^q(\mathcal {O};\mathbb {R}^2)$, see e.g. [43, Subsection 7.4]. Let $f\in L^q(\mathcal {O};\mathbb {R}^2)$ and let $\psi _f\in W^{1,q}(\mathcal {O})$ be the unique solution to the following elliptic problem

2.1 $$\begin{aligned} \left\{ \begin{aligned} \Delta \psi _f&= {{\,\textrm{div}\,}}f\quad&\text { on }&\mathcal {O},\\ \partial _n \psi _f&= f\cdot n&\text { on }&\Gamma _u \cup \Gamma _b. \end{aligned} \right. \end{aligned}$$

Here *n* denotes the exterior normal vector field on $\partial \mathcal {O}$. Of course, the above elliptic problem is interpret in its natural weak formulation:

2.2 $$\begin{aligned} \int _{\mathcal {O}} \nabla \psi _f \cdot \nabla \varphi \,\textrm{d}x\textrm{d}z = \int _{\mathcal {O}} f\cdot \nabla \varphi \,\textrm{d}x \textrm{d}z\ \ \text { for all } \varphi \in C^{\infty }(\mathcal {O}). \end{aligned}$$

By [43, Corollary 7.4.4], we have $\psi _f\in W^{1,q}(\mathcal {O})$ and $\Vert \nabla \psi _f\Vert _{L^{q}(\mathcal {O};\mathbb {R}^2)}\lesssim \Vert f\Vert _{L^q(\mathcal {O};\mathbb {R}^2)}$ (the proof of such estimate can also be obtained by the Lax-Milgram theorem in Banach spaces [35, Theorem1.1], see also the proof of Theorem 2.2 below). Then the Helmholtz projection is given by $\mathbb {P}_q$ is defined as

$$\begin{aligned} \mathbb {P}_q f= f- \nabla \psi _f, \quad f\in L^q(\mathcal {O};\mathbb {R}^2). \end{aligned}$$

Next we define the Stokes operator on $L^q(\mathcal {O};\mathbb {R}^2)$. For convenience of notation, we actually define $A_q$ as minus the Stokes operator so that $A_q$ is a positive operator for $q=2$ (i.e. $\langle A_2 u,u \rangle \ge 0$ for all $u\in \textsf{D}(A_2)$). Let $\mathbb {L}^q:=\mathbb {P}(L^q(\mathcal {O};\mathbb {R}^2))$. Then, we define the operator $A_q:\textsf{D}(A_q)\subseteq \mathbb {L}^q\rightarrow \mathbb {L}^q$ where

$$\begin{aligned} \textsf{D}(A_q)= \big \{f=(f_1,f_2)\in W^{2,q}(\mathcal {O};\mathbb {R}^2)\cap \mathbb {L}^q\,:\, \ {}&f|_{\Gamma _b}=0, \\&f_2 |_{\Gamma _u}=\partial _z f_1|_{\Gamma _u}=0\big \}, \end{aligned}$$

and $$A_q u=-\mathbb {P}_q \Delta u$$ for $$u\in \textsf{D}(A_q)$$.

In the main arguments we need stochastic maximal $L^q$-regularity estimates for stochastic convolutions. By [53] (see also [2, 52]), it is enough to show the boundedness of the $H^{\infty }$-calculus for $A_q$. For the main notation and basic results on the $H^{\infty }$-calculus we refer to [43, Chapters 3 and 4] and [32, Chapter 10].

In the following, we let

$$\begin{aligned} \mathbb {H}^{s,q}(\mathcal {O}):=H^{s,q}(\mathcal {O};\mathbb {R}^2)\cap \mathbb {L}^q, \ \ s\in \mathbb {R}. \end{aligned}$$

#### Theorem 2.1

(Boundedness $H^{\infty }$-calculus) For all $q\in (1,\infty )$, the operator $A_q$ is invertible and has a bounded $H^{\infty }$-calculus of angle $<\frac{\pi }{2}$. Moreover the domain of the fractional powers of $A_q$ is characterized as follows:

1. $$\textsf{D}(A_q^{s})= \mathbb {H}^{2s,q}(\mathcal {O})$$ if $$0\le s<\frac{1}{2q}$$.
2. $$\textsf{D}(A_q^{s})= \big \{f\in \mathbb {H}^{2\,s,q}(\mathcal {O})\,|\, f|_{\Gamma _b} =0,\ f_2|_{\Gamma _u}=0 \big \} $$ if $$\frac{1}{2q}<s<\frac{1}{2}+\frac{1}{2q}$$.
3. $$ \textsf{D}(A_q^{s})= \big \{f\in \mathbb {H}^{2\,s,q}(\mathcal {O})\,|\, f|_{\Gamma _b} =0,\ f_2|_{\Gamma _u}=\partial _z f_1|_{\Gamma _u}=0 \big \} $$ if $$\frac{1}{2}+\frac{1}{2q}<s<1$$.

The above implies that $-A_q$ generates an analytic semigroup on $\mathbb {L}^q$.

For convenience of notation, we will simply write *A* in place of $A_2$. Moreover we define

$$\begin{aligned} H:=\mathbb {L}^2,\quad V:=\textsf{D}(A^{1/2}),\quad \mathcal {D}(\mathcal {O}):=\{f\in C^{\infty }_{\textrm{c}}(\mathcal {O};\mathbb {R}^2)\, :\, {{\,\textrm{div}\,}}f=0\}. \end{aligned}$$

We denote by $\langle \cdot ,\cdot \rangle$ and $\Vert \cdot \Vert$ the inner product and the norm in *H* respectively. In the sequel we will denote by $V^{*}$ the dual of *V* and we will identify *H* with $H^{*}$. Every time *X* is a reflexive Banach space such that the embedding $X\hookrightarrow H$ is continuous and dense, denoting by $X^*$ the dual of *X*, the scalar product $\left\langle \cdot ,\cdot \right\rangle$ in *H* extends to the dual pairing between *X* and $X^{*}$. We will simplify the notation accordingly.

Theorem 2.1 could be known to experts. For the reader’s convenience, we provide in “Appendix A” a complete and relatively short proof based on the recent strategy used in [42] for the $H^{\infty }$-calculus for the Stokes operator on Lipschitz domains [42, Theorem16].

### The Neumann map

Now we are interested in $L^q$-estimates for the Neumann map, i.e. we are interested in studying the weak solutions of the elliptic problem

2.3 $$\begin{aligned} \left\{ \begin{aligned} -\Delta u+\nabla \pi&=0,&\text { on }&\mathcal {O},\\ {{\,\textrm{div}\,}}u&=0,\qquad&\text { on }&\mathcal {O}, \\ u( \cdot ,0)&=0,&\text { on }&\Gamma _b,\\ \partial _{z}u_1( \cdot ,a)&=g,&\text { on }&\Gamma _u,\\ u_2&=0,&\text { on }&\Gamma _u. \end{aligned}\right. \end{aligned}$$

To state the main result of this subsection, we need to formulate (2.3) in the weak setting. To this end, we argue formally. Take $\varphi =(\varphi _1,\varphi _2)\in C^{\infty }(\mathcal {O};\mathbb {R}^2)$ such that ${{\,\textrm{div}\,}}\varphi =0$,

$$\begin{aligned} \varphi (\cdot ,0)=0, \quad \text { and } \quad \varphi _2( \cdot ,a) =0. \end{aligned}$$

A formal integration by parts shows that (2.3) implies

2.4 $$\begin{aligned} \int _{\mathcal {O}} \nabla u: \nabla \varphi \,\textrm{d}x \textrm{d}z= -\int _{\mathbb {T}} g(x) \varphi _1(x,a)\,\textrm{d}x. \end{aligned}$$

In particular, the RHS of (2.4) makes sense even in case *g* is a distribution if we interpret $\int _{\mathbb {T}} g(x) \varphi _1(x,a)\,\textrm{d}x =\langle \varphi _1(\cdot ,a),g\rangle$.

#### Theorem 2.2

Let $q\in (1,\infty )$, for all $g\in W^{-1/q,q}(\Gamma _u)$ there exists a unique $(u,\pi )\in W^{1,q}(\mathcal {O};\mathbb {R}^2)\times L^q(\mathcal {O})/ \mathbb {R}$ weak solution of (2.3). Moreover $(u,\pi )$ satisfy

2.5 $$\begin{aligned} \Vert u \Vert _{W^{1,q}(\mathcal {O};\mathbb {R}^2)}+\Vert \pi \Vert _{L^{q}(\mathcal {O})/{\mathbb {R}}}\le C\Vert g\Vert _{W^{-1/q,q}(\Gamma _u)}. \end{aligned}$$

Finally, if $g\in W^{1-1/q,q}(\Gamma _u)$, then $(u,\pi )\in W^{2,q}(\mathcal {O};\mathbb {R}^2)\times W^{1,q}(\mathcal {O})/ \mathbb {R}$ and

2.6 $$\begin{aligned} \Vert u \Vert _{W^{2,q}(\mathcal {O};\mathbb {R}^2)}+\Vert \pi \Vert _{W^{1,q}(\mathcal {O})/{\mathbb {R}}}\le C\Vert g\Vert _{W^{1-1/q,q}(\Gamma _u)}. \end{aligned}$$

#### Proof

We divide the proof into three steps.

*Step 1: Proof of *(2.5). Let $A_q$ be as in Sect. 2. We prove (2.5) by applying the Lax-Milgram theorem of [35, Theorem 1.1] to the form $a:Y_1\times Y_2\rightarrow \mathbb {R}$ where

$$\begin{aligned} a(u,\varphi )= \int _{\mathcal {O}} \nabla u: \nabla \varphi \,\textrm{d}x\textrm{d}z,\quad Y_1=\textsf{D}(A_q^{1/2}), \quad Y_2=\textsf{D}(A_{q'}^{1/2}). \end{aligned}$$

Recall that, by Theorem 2.1,

$$\begin{aligned} \textsf{D}(A_q^{1/2})=\{v=(v_1,v_2)\in \mathbb {H}^{1,q}(\mathcal {O}):\, v|_{\Gamma _b}=0,\ v_2|_{\Gamma _u}=0\}. \end{aligned}$$

Since $W^{1,q'}(\mathcal {O})\ni \varphi \mapsto \varphi _1|_{\Gamma _u}\in W^{1-1/q',q'}(\Gamma _u)= W^{1/q,q'}(\Gamma _u)$, we have

2.7 $$\begin{aligned} |\langle \varphi _1(\cdot ,a),g\rangle |\le \Vert g\Vert _{W^{-1/q,q}(\Gamma _u)}\Vert \varphi \Vert _{W^{1/q,q'}(\Gamma _u)} \lesssim \Vert g\Vert _{W^{-1/q,q}(\Gamma _u)}\Vert \varphi \Vert _{W^{1,q'}(\mathcal {O})}.\qquad \end{aligned}$$

Hence the Lax–Milgram theorem of of [35, Theorem1.1] implies the existence of *u* as in (2.5) provided, for all $v\in \textsf{D}(A_p^{1/2})$,

2.8 $$\begin{aligned} \Vert \nabla v\Vert _{L^q(\mathcal {O};\mathbb {R}^2)}\eqsim \sup \Big \{\int _{\mathcal {O}} \nabla v:\nabla f \,\textrm{d}x\textrm{d}z\, \Big |\,f\in \textsf{D}(A^{1/2}_{q'})\text { and } \Vert f\Vert _{\textsf{D}(A^{1/2}_{q'})}\le 1 \Big \}.\nonumber \\ \end{aligned}$$

The case $> rsim$ of (2.8) follows from the Hölder inequality. To prove the opposite inequality, we argue by duality. We start by discussing some known facts about the “Sobolev tower” of spaces associated the operator $A_p$:

$$\begin{aligned} X_{\alpha ,A_q}&= \textsf{D}(A_p^{\alpha }) \&\text { for }&\alpha \ge 0,\\ X_{\alpha ,A_q}&= (\mathbb {L}^q,\Vert A_q^{\alpha } \cdot \Vert _{\mathbb {L}^q})^{\sim } \ {}&\text { for }&\alpha <0. \end{aligned}$$

Here $\sim$ denotes the completion (since $0\in \rho (A_q)$ by Theorem 2.1, we have that $f\mapsto \Vert A_q^{\alpha } f\Vert _{\mathbb {L}^q}$ is a norm for all $\alpha <0$). Since $(A_q)^*=A_{q'}$, it follows that (see e.g. [5, Chapter 5, Theorem 1.4.9])

2.9 $$\begin{aligned} (X_{\alpha ,A_q})^*= X_{-\alpha ,A_{q'}}. \end{aligned}$$

Now we can proceed in the proof of $\lesssim$ in (2.8). Firstly, as $\textsf{D}(A_q)\ \hookrightarrow \textsf{D}(A^{1/2}_q)$ is dense for all $q\in (1,\infty )$, we can prove such inequality assuming $v\in \textsf{D}(A_q)$. In the latter case, the duality (2.9) and the Hahn–Banach theorem imply the existence of $g\in X_{-\alpha ,A_{q'}}$ of unit norm such that

$$\begin{aligned} \Vert A_q^{1/2} v\Vert _{L^q(\mathcal {O};\mathbb {R}^2)}&=\int _{\mathcal {O}} A^{1/2}_q v \cdot A_{q'}^{-1/2} g \,\textrm{d}x\textrm{d}z\\&{\mathop {=}\limits ^{(i)}}\int _{\mathcal {O}} A_q v \cdot A_{q'}^{-1} g \,\textrm{d}x \textrm{d}z\\&{\mathop {=}\limits ^{(ii)}}-\int _{\mathcal {O}} \Delta v \cdot A_{q'}^{-1} g \,\textrm{d}x \textrm{d}z\\&{\mathop {=}\limits ^{(iii)}}-\int _{\mathcal {O}} \nabla v : \nabla (A^{-1}_{q'} g) \,\textrm{d}x \textrm{d}z \end{aligned}$$

where in (i) we used that $A_q^{1/2}v =A_{q}^{-1/2} (A_q v)$ and $(A_{q}^{-1/2})^*=A_{q'}^{-1/2}$, in (ii) that $A_q=-\mathbb {P}_q \Delta _q$ and therefore $\mathbb {P}_{q'}A_{q'}^{-1} g=A_{q'}^{-1} g$ as $A_{q'}^{-1} g\in \textsf{D}(A^{1/2}_{q'})\subseteq \mathbb {L}^{q'}(\mathcal {O})$. Finally, in (iii) we used that no boundary terms appear due to the boundary conditions and $\partial _z v_1(\cdot ,a)=0$ as $v\in \textsf{D}(A_q)$.

Hence the case $\lesssim$ of (2.8) follows from the above chain of equality, the fact that $\textsf{D}(A_q^{1/2})\hookrightarrow W^{1,q}(\mathcal {O};\mathbb {R}^2)$ and $A^{-1}_{q'}:X_{-1/2,A_{q'}}\rightarrow X_{1/2,A_{q'}}$ is an isomorphism.

Now, the existence of the pressure $\pi$ satisfying the estimate (2.5) is standard and follows from the De Rham theorem, see e.g. [29, Corollary III.5.1, Lemma IV.1.1].

*Step 2: Proof of* (2.6). By Step 1, it suffices to prove the existence of a solution $(u,\pi )\in W^{2,q}(\mathcal {O})\times W^{1,q}(\mathcal {O})/\mathbb {R}$ for which (2.3) holds. In case of $g\in C^{\infty }(\Gamma _u)$, the conclusion follows from standard $L^2$-theory and we will present the argument in this case at the end of the proof. In the remaining case we argue by density. Note that, arguing as in the proof of Proposition A.4, a localization argument and [43, Theorem 7.2.1] (applied with time as a dummy variable) yield the following a-priori estimates for solutions $(u,\pi )\in W^{2,q}(\mathcal {O};\mathbb {R}^2)\times W^{1,q}(\mathcal {O})/\mathbb {R}$ to (2.3):

$$\begin{aligned} \Vert u\Vert _{ W^{2,q}(\mathcal {O};\mathbb {R}^2)}+ \Vert \nabla \pi \Vert _{ W^{1,q}(\mathcal {O};\mathbb {R}^2)}\le C\Vert u\Vert _{W^{2-2/q,q}(\mathcal {O};\mathbb {R}^2)}+ C\Vert g\Vert _{W^{1-1/q,q}(\Gamma _u)}&\\ \le \varepsilon \Vert u\Vert _{W^{2,q}(\mathcal {O};\mathbb {R}^2)}+C_{\varepsilon }\Vert u\Vert _{W^{1,q}(\mathcal {O};\mathbb {R}^2)} + C\Vert g\Vert _{W^{1-1/q,q}(\Gamma _u)}&\\ \le \varepsilon \Vert u\Vert _{W^{2,q}(\mathcal {O};\mathbb {R}^2)}+C_{\varepsilon }\Vert g\Vert _{W^{1-1/q,q}(\Gamma _u)}&, \end{aligned}$$

where $\varepsilon >0$ is arbitrary and in the last step we applied Step 1.

The above shows $\Vert u\Vert _{ W^{2,q}(\mathcal {O};\mathbb {R}^2)}+ \Vert \nabla \pi \Vert _{ W^{1,q}(\mathcal {O})}\lesssim \Vert g\Vert _{W^{1-1/q,q}(\mathbb {T})}$ for all solutions $(u,\pi )\in W^{2,q}(\mathcal {O};\mathbb {R}^2)\times W^{1,q}(\mathcal {O})/\mathbb {R}$ to (2.3). Combining this, the density of $C^{\infty }(\Gamma _u)$ in $W^{1-1/q,q}(\Gamma _u)$, and the above mentioned solvability for $g\in C^{\infty }(\Gamma _u)$; one readily obtains the existence of solutions to (2.3) in the class $W^{2,q}(\mathcal {O})\times W^{1,q}(\mathcal {O})/\mathbb {R}$.

*Step 3: Proof of the regularity of* $(u,\pi )$ *in case of* $g\in C^{\infty }(\mathbb {T})$. The proof of this fact follows the lines of Proposition A.2. First, by Lax-Milgram Lemma and [51, Proposition 1.1, Proposition 1.2], there exists a unique couple, $(u,\pi )\in V\times L^2(\mathcal {O})$ such that

2.10 $$\begin{aligned} \int _{\mathcal {O}} \nabla u:\nabla \phi \,\textrm{d}x\textrm{d}z&=-\int _{{\mathbb {T}}} g(x)\phi _1(x,a)\,\textrm{d}x \quad \forall \phi \in V \end{aligned}$$

2.11 $$\begin{aligned} \langle -\Delta u+\nabla \pi ,\varphi \rangle&=0 \quad \forall \varphi \in C^{\infty }_c(\mathcal {O};\mathbb {R}^2) \end{aligned}$$

2.12 $$\begin{aligned} \Vert u\Vert _V+\Vert \pi \Vert _{L^2/\mathbb {R}}&\lesssim \Vert g\Vert _{H^{-1/2}(\Gamma _u)}. \end{aligned}$$

Now, let us fix $h>0$, extend periodically either *u* and *g* in the *x* direction and consider $\phi =\tau _{h}\tau _{-h} u$ as a test function in (2.10), where $\tau _h v=\frac{v(x+h,z)-v(x,z)}{h}.$ Then by change of variables, it follows that

$$\begin{aligned} \Vert \tau _h \nabla u\Vert _{L^2(\mathcal {O})}^2&\le C \Vert \tau _{h} g\Vert _{L^2(\Gamma _u)} \Vert \tau _{h} u\Vert _{L^2(\Gamma _u)}\\ {}&\le C \Vert \tau _{h} g\Vert _{L^2(\Gamma _u)}\Vert \tau _{h} u\Vert _{L^2(\mathcal {O})}+C \Vert \tau _{h} g\Vert _{L^2(\Gamma _u)}\Vert \tau _{h} \nabla u\Vert _{L^2(\mathcal {O})}\\ {}&\le C\Vert g\Vert _{C^1(\Gamma _u)}\Vert \tau _h u\Vert _{L^2(\mathcal {O})}+\frac{\Vert \tau _{h} \nabla u\Vert _{L^2(\mathcal {O})}^2}{2}+C\Vert g\Vert _{C^1(\Gamma _u)}^2. \end{aligned}$$

Therefore

2.13 $$\begin{aligned} \Vert \tau _h \nabla u\Vert _{L^2(\mathcal {O})}^2\le C\Vert g\Vert _{C^1(\Gamma _u)}\Vert \tau _h u\Vert _{L^2(\mathcal {O})}+C\Vert g\Vert _{C^1(\Gamma _u)}^2. \end{aligned}$$

Since $u\in V$ and (2.12) holds the right hand side of inequality (2.13) is uniformly bounded in $h\rightarrow 0$ and this implies

2.14 $$\begin{aligned} \Vert \partial _x \nabla u\Vert _{L^2(O)}^2\le C\Vert g\Vert _{C^1(\Gamma _u)}^2. \end{aligned}$$

Let us now consider $\phi =\partial _x\psi ,\ \psi \in \mathcal {D}(\mathcal {O})$ as test function in (2.10). Thanks to [51, Proposition 1.1, Proposition 1.2], $\partial _x \pi \in L^2(\mathcal {O})$ and $\Vert \partial _x\pi \Vert _{L^2}\lesssim \Vert g\Vert _{C^1(\Gamma _u)}$. Since *u* is divergence free and (2.11) holds, then

$$\begin{aligned} \partial _z \pi =\partial _{xx}u_2-\partial _{xz}u_1\in L^2(\mathcal {O}). \end{aligned}$$

Therefore $\Vert \nabla \pi \Vert _{L^2}\lesssim \Vert g\Vert _{C^1(\Gamma _u)}.$ Lastly, again by relation (2.11)

$$\begin{aligned} \partial _{zz}u_1=\partial _x\pi -\partial _{xz}u_1\in L^2(\mathcal {O}) \end{aligned}$$

Combining all the information obtained we get

$$\begin{aligned} \Vert u\Vert _{H^{2}(\mathcal {O};\mathbb {R}^2)}+\Vert \pi \Vert _{H^1(\mathcal {O})/\mathbb {R}}\le C\Vert g\Vert _{C^1(\Gamma _u)}^2 \end{aligned}$$

Iterating the argument one gets that $(u,\pi )\in H^{k+1}(\mathcal {O};\mathbb {R}^2)\times H^{k}(\mathcal {O})$ provided $g\in C^k(\Gamma _u)$ for all $k\ge 1$. Now the claim of Step 3 follows from Sobolev embeddings. $\square$

Next we denote by $\mathcal {N}$ the solution map defined by Theorem 2.2 which associate to a boundary datum *g* the velocity *u* solution of (2.3), i.e. $\mathcal {N}g:=u$. From the above result we obtain

#### Corollary 2.3

Let $\mathcal {N}$ and $\mathcal {H}$ be the Neumann map and a Hilbert space, respectively. Then, for all $q\ge 2$ and $\varepsilon >0$,

1. labelit:mappingspsNM1 $\mathcal {N}\in \mathscr {L}(W^{-\alpha ,q}(\Gamma _u;\mathcal {H});\gamma (\mathcal {H},\textsf{D}(A_q^{\frac{1-\alpha }{2}+\frac{1}{2q}-\varepsilon })))$ for $\alpha \in [0,\frac{1}{q}]$.
2. $$\mathcal {N}\in \mathscr {L}(L^q({\Gamma _u};\mathcal {H});\gamma (\mathcal {H},\textsf{D}(A_q^{\frac{1}{2}+\frac{1}{2q}-\varepsilon })))$$.

#### Proof

To begin, recall that $W^{s,q}(\Gamma _u;\mathcal {H})=\gamma (\mathcal {H},W^{s,q}(\Gamma _u))$ for all $s\in \mathbb {R}$ and $q\in (1,\infty )$, see Sect. 1.1.1. Hence, due to the ideal property of $\gamma$-radonifying operators [32, Theorems 9.1.10 and 9.1.20], it is enough to consider the scalar case $\mathcal {H}=\mathbb {R}$.

(1): By interpolating with the real method $(\cdot ,\cdot )_{\theta ,q}$ where $\theta \in (0,1)$ (see e.g. [10, Theorem 6.4.5]), the estimates in Theorem 2.2 yield

$$\begin{aligned} \mathcal {N}: W^{\theta -1/q,q} (\Gamma _u)\rightarrow W^{\theta +1,q}(\mathcal {O})\ \ \text { for all }\theta \in (0,1). \end{aligned}$$

Moreover, by construction $\mathcal {N}[u]$ satisfies

$$\begin{aligned} \mathcal {N}[u]|_{\Gamma _b}=0, \quad \text { and }\quad (\mathcal {N}[u])_2 |_{\Gamma _u}=0, \end{aligned}$$

where $(\cdot )_2$ denotes the second component. Hence (1) follows from the description of the fractional power of $A_q$ in Theorem 2.1 and that $B^{1+\theta }_{q,q}(\mathcal {O};\mathbb {R}^2)\hookrightarrow H^{\theta +1-\varepsilon ,q}(\mathcal {O};\mathbb {R}^2)$.

(2): Follows from (1) and $L^q(\Gamma _u)\hookrightarrow B^{0}_{q,q}(\Gamma _u)$ as $q\ge 2$. $\square$

### Deterministic Navier–Stokes equations

Let us consider the deterministic Navier–Stokes equations with homogeneous boundary conditions

2.15 $$\begin{aligned} \left\{ \begin{aligned} \partial _{t}\overline{u}+\overline{u}\cdot \nabla \overline{u}+\nabla \overline{\pi }&=\Delta \overline{u}+\overline{f}, \quad&\text { on }&(0,T)\times \mathcal {O},\\ {\text {div}}\overline{u}&=0,&\text { on }&(0,T)\times \mathcal {O},\\ \overline{u}&= 0,&\text { on }&(0,T)\times \Gamma _b,\\ \partial _{z}\overline{u}_1&=0,&\text { on }&(0,T)\times \Gamma _u,\\ \overline{u}_2&=0,&\text { on }&(0,T)\times \Gamma _u, \\ \overline{u}(0)&=\overline{u}_0,&\text { on }&\mathcal {O}. \end{aligned} \right. \end{aligned}$$

Define the trilinear form $b:\mathbb {L}^{4}\times V\times \mathbb {L} ^{4}\rightarrow \mathbb {R}$ as

2.16 $$\begin{aligned} b\left( u,v,w\right) =\sum _{i,j=1}^{2}\int _{\mathcal {O}}u_{i} \partial _{i}v_{j} w_{j} \, \textrm{d}x\textrm{d}z=\int _{\mathcal {O}}\left( u\cdot \nabla v\right) \cdot w\,\textrm{d}x\textrm{d}z \end{aligned}$$

which is well-defined and continuous on $\mathbb {L}^{4}\times V\times \mathbb {L}^{4}$ by the Hölder inequality. Since by Sobolev embedding theorem $V\subset \mathbb {L}^{4}$, *b* is also defined and continuous on $V\times V\times V$. Moreover, by standard interpolation inequalities,

2.17 $$\begin{aligned} \Vert f\Vert _{L^{4}\left( \mathcal {O}\right) }^{2}\le C\Vert f\Vert _{L^{2}\left( \mathcal {O}\right) }\Vert f\Vert _{H^{1}\left( \mathcal {O}\right) } \ \ \text { for all }f\in H^{1}\left( \mathcal {O}\right) . \end{aligned}$$

Integrating by parts, the standard oddity relation below holds

$$\begin{aligned} b\left( u,v,w\right) =-b\left( u,w,v\right) \end{aligned}$$

if $$u\in \mathbb {L}^{4}$$, $$v,w\in V$$.

Lastly we introduce the operator

$$\begin{aligned} B:\mathbb {L}^{4}\times \mathbb {L}^{4}\rightarrow V^* \end{aligned}$$

defined by the identity

$$\begin{aligned} \left\langle B\left( u,v\right) ,\phi \right\rangle =-b\left( u,\phi ,v\right) =-\int _{\mathcal {O}}\left( u\cdot \nabla \phi \right) \cdot v\,\textrm{d}x\textrm{d}z \end{aligned}$$

for all $\phi \in V$. When $v\in V$, we may also write

$$\begin{aligned} \left\langle B\left( u,v\right) ,\phi \right\rangle =b( u,v,\phi ).\end{aligned}$$

Moreover, when $u\cdot \nabla v\in L^{2}\left( \mathcal {O};\mathbb {R}^{2}\right)$, it is explicitly given by

$$\begin{aligned} B\left( u,v\right) =\mathbb {P}( u\cdot \nabla v). \end{aligned}$$

We have to define our notion of weak solution for problem (2.15).

#### Definition 2.4

Given $\overline{u}_{0}\in H$ and $\overline{f}\in L^{2}\left( 0,T;V^{*}\right)$, we say that

$$\begin{aligned} \overline{u}\in C\left( \left[ 0,T\right] ;H\right) \cap L^{2}\left( 0,T;V\right) \end{aligned}$$

is a weak solution of equation (2.15) if for all $\phi \in \textsf{D}\left( A\right)$ and $t\in [0,T]$,

$$\begin{aligned}&\left\langle \overline{u}\left( t\right) ,\phi \right\rangle -\int _{0}^{t}b\left( \overline{u}\left( s\right) ,\phi ,\overline{u}\left( s\right) \right) \,\textrm{d}s\\&\quad =\left\langle \overline{u}_{0},\phi \right\rangle -\int _{0}^{t}\left\langle \overline{u}\left( s\right) ,A\phi \right\rangle \,\textrm{d}s+\int _{0}^{t}\left\langle \overline{f}\left( s\right) ,\phi \right\rangle _{V^*,V} \,\textrm{d}s. \end{aligned}$$

The following results are simple adaptations of classical results, see for instance [27, 37, 50, 51].

#### Lemma 2.5

If $$u,v\in L^{4}\left( 0,T;\mathbb {L}^{4}\right) $$ then

2.18 $$\begin{aligned} B\left( u,v\right) \in L^{2}\left( 0,T;V^*\right) . \end{aligned}$$

Moreover,

2.19 $$\begin{aligned}{} & {} |b\left( u,v,w\right) |\le \varepsilon \Vert v\Vert _{V}^{2}+\varepsilon '\Vert u\Vert _{V}^{2}+\frac{C }{\varepsilon ^2\varepsilon '}\Vert u\Vert ^{2}\Vert w\Vert _{\mathbb {L}^4}^{4} \end{aligned}$$

2.20 $$\begin{aligned}{} & {} |b\left( u,v,w\right) |\le \varepsilon \Vert v\Vert _{V}^{2}+\varepsilon '\Vert w\Vert _{V}^{2}+\frac{C }{\varepsilon ^2\varepsilon '}\Vert w\Vert ^{2}\Vert u\Vert _{\mathbb {L}^4}^{4}, \end{aligned}$$

where *C* is a constant independent of $\varepsilon$ and $\varepsilon '$.

#### Theorem 2.6

For every $\overline{u}_{0}\in H$ and $\overline{f}\in L^{2}\left( 0,T;V^*\right)$ there exists a unique weak solution of Eq. (2.15). It satisfies

$$\begin{aligned} \Vert \overline{u}\left( t\right) \Vert ^{2}+2\nu \int _{0} ^{t}\Vert \nabla \overline{u}\left( s\right) \Vert _{L^{2}}^{2}\,\textrm{d}s=\Vert \overline{u}_{0}\Vert ^{2}+2\int _{0}^{t}\left\langle \overline{u}\left( s\right) ,\overline{f}\left( s\right) \right\rangle _{V^*,V} \,\textrm{d}s. \end{aligned}$$

If $\left( \overline{u}_{0}^{n}\right) _{n\in \mathbb {N}}$ is a sequence in *H* converging to $\overline{u}_{0}\in H$ and $\left( \overline{f}^{n}\right) _{n\in \mathbb {N}}$ is a sequence in $L^{2}\left( 0,T;V^*\right)$ converging to $\overline{f}\in L^{2}\left( 0,T;V^*\right)$, then the corresponding unique solutions $\left( \overline{u}^{n}\right) _{n\in \mathbb {N}}$ converge to the corresponding solution $\overline{u}$ in $C\left( \left[ 0,T\right] ;H\right)$ and in $L^{2}\left( 0,T;V\right)$.

### Stochastic maximal $L^p$-regularity

Let $\mathcal {H}$ and $(W_{\mathcal {H}}(t))_{t\ge 0}$ be a Hilbert space and a cylindrical $\mathcal {F}-$Brownian motion on $\mathcal {H}$, respectively. The following result was proven in [53], see also [52, Section 7] and [9, Section 3] for additional references. Below, for a Banach space *Y*, $H^{s,q}(\mathbb {R}_+;Y)$ denotes the *Y*-valued Bessel potential space on $\mathbb {R}_+$ with smoothness $s\in \mathbb {R}$ and integrability *q*; such space can be defined either by complex interpolation (see e.g. [43, Chapter 3, Section4.5]) or by restriction from $\mathbb {R}$ (see e.g. [3, Subsection 3.1]). For the notion of $H^{\infty }$-calculus and $\gamma$-radonifying operators $\gamma (\mathcal {H},Y)$ we refer to [32, Chapter 9 and10].

#### Theorem 2.7

Let *X* be a Banach space isomorphic to a closed subspace of $L^q(D,\mu )$ where $q\in [2,+\infty )$ and $(D,\mathscr {A},\mu )$ is a $\sigma$-finite measure space. Let $\mathcal {A}$ be an invertible operator and assume that it admits a bounded $H^{\infty }$ calculus of angle $<\pi /2$ on *X* and let $(\mathcal {S}(t))_{t\ge 0}$ the bounded analytic semigroup generated by $-\mathcal {A}$. For all $\mathcal {F}-$adapted $G\in L^p(\mathbb {R}_+\times \Omega ;\gamma (\mathcal {H};X))$ the stochastic convolution process

$$\begin{aligned} U(t)=\int _0^t \mathcal {S}(t-s)G(s)\,\textrm{d}W_{\mathcal {H}}(s)\quad t\ge 0, \end{aligned}$$

is well defined in *X*, takes values in the fractional domain $\textsf{D}(\mathcal {A}^{1/2})$ almost surely and for all $2<p<+\infty$ the following space-time regularity estimate holds: $\forall \theta \in [0,\frac{1}{2})$

2.21 $$\begin{aligned} \textbf{E}\left[ \Vert U(t) \Vert ^p_{H^{\theta ,p}(\mathbb {R}_+;\textsf{D}(\mathcal {A}^{1/2-\theta }))} \right] \le C_{\theta }^p\textbf{E}\left[ \Vert G\Vert _{L^p(\mathbb {R}_+;L^q(O;\mathcal {H}))}^p\right] \end{aligned}$$

with a constant $C_{\theta }$ independent of *G*.

For extensions of the above result we refer to [2, 39] for the weighted case, and to [3, Subsection 6.2] for the case of homogeneous spaces. However, the latter situations will not be considered here.

## Well-posedness

### Stokes equations

As discussed in Sect. 1.1, we start by considering the linear problem (1.3). According to [18, 19], the mild solution *w* of the former problem is formally given by

3.1 $$\begin{aligned} w(t)=A_q\int _0^t S_q(t-s)\mathcal {N}[h_b(s)]\,\textrm{d}W_{\mathcal {H}}(s). \end{aligned}$$

Here $A_q$ is (minus) the Stokes operator with homogeneous boundary conditions, and $(S_q(t))_{t\ge 0}$ its corresponding semigroup (cf.Theorem 2.1).

Next step is to prove that *w*(*t*) is well defined in some functional spaces and has some regularities useful to treat the nonlinearity of the Navier–Stokes equations.

#### Proposition 3.1

Let $\alpha \in [0,\frac{1}{q}]$ and assume that $h_b:(0,T)\times \Omega \rightarrow W^{-\alpha ,q}(\Gamma _u;\mathcal {H})$ is $\mathcal {F}$-progressive measurable with $\textbf{P}-a.s.$ paths in $L^p(0,T;W^{-\alpha ,q}(\Gamma _u))$. Then the process *w* defined in (3.1) is a well defined process with $\textbf{P}-a.s.$ paths in

$$\begin{aligned} H^{\theta ,p}(0,T;\textsf{D}(A_q^{\frac{1}{2q}-\frac{\alpha }{2}-\theta -\varepsilon })) \ \ \text { for all }\ \theta \in [0,\tfrac{1}{2}),\ \varepsilon >0. \end{aligned}$$

In particular, if $h_b$ satisfies Hypothesis 1.1, then *w* has $\textbf{P}-a.s.$ trajectories in $C([0,T];H)\cap L^4(0,T;\mathbb {L}^4)$.

#### Proof

By replacing $h_b$ by $\varvec{1}_{[0,\tau _n]\times \Omega }h_{b}$, $\tau _n$ being the following stopping time

$$\begin{aligned} \tau _n:=\{t\in [0,T]:\, \Vert h\Vert _{L^p(0,t;W^{-\alpha ,q}(\Gamma _u;\mathcal {H}))}\ge n\} \quad \text { where }\quad \inf \varnothing :=T, \end{aligned}$$

for all $n\ge 1$, it is enough to consider the case $h_b\in L^p((0,T)\times \Omega ;W^{-\alpha ,q}(\Gamma _u;\mathcal {H}))$.

Let $\varepsilon >0$ be fixed later. From Corollary 2.3 and Theorem 2.7 we have that $\textbf{P}-a.s.$ and for each $\theta \in [0,\frac{1}{2})$

$$\begin{aligned} \widetilde{w}=\int _0^\cdot S_q(\cdot -s)A_q^{\frac{1-\alpha }{2}+\frac{1}{2q}-\varepsilon }\mathcal {N}[h_b(s) ]\,\textrm{d}W_{\mathcal {H}}(s)\in H^{\theta ,p}(0,T;\textsf{D}(A_q^{1/2-\theta })) \quad \end{aligned}$$

Therefore, $$\textbf{P}-a.s.$$,

$$\begin{aligned} w=A_q^{\frac{1+\alpha }{2}-\frac{1}{2q}+\varepsilon }\widetilde{w}\in H^{\theta ,p}(0,T;\textsf{D}(A_q^{\frac{1}{2q}-\frac{\alpha }{2}-\theta -\varepsilon })). \end{aligned}$$

Finally, note that, by Hypothesis 1.1, Theorem 2.1 and the Sobolev embeddings (see e.g. [9, Proposition 2.7]) we can find $\theta _1,\theta _2\in [0,\frac{1}{2})$ and $\varepsilon >0$ such that

$$\begin{aligned} H^{\theta _1,p}(0,T;\textsf{D}(A_q^{\frac{1}{2q}-\frac{\alpha }{2}-\theta _1-\varepsilon }))&\hookrightarrow C([0,T];H),\\ H^{\theta _2,p}(0,T;\textsf{D}(A_q^{\frac{1}{2q}-\frac{\alpha }{2}-\theta _2-\varepsilon }))&\hookrightarrow L^4(0,T;\mathbb {L}^4). \end{aligned}$$

where the first embedding follows from $\alpha <\frac{1}{q}-\frac{2}{p}$ and the second one from the remaining conditions in Hypothesis 1.1. Hence the proof is complete. $\square$

### Auxiliary Navier–Stokes type equations

Having solved the Stokes problem we introduce the auxiliary variable

$$\begin{aligned} v\left( t\right) =u\left( t\right) -w( t), \end{aligned}$$

which satisfies (1.4), i.e.

$$\begin{aligned} \left\{ \begin{aligned} \partial _{t}v+\left( v+w\right) \cdot \nabla \left( v+w\right) +\nabla \left( P-\rho \right)&=\nu \Delta v,&\text { on }&(0,T)\times \mathcal {O},\\ {\text {div}}v&=0,&\text { on }&(0,T)\times \mathcal {O},\\ v&= 0,&\text { on }&(0,T)\times \Gamma _b,\\ \partial _{z}v_1&=0,&\text { on }&(0,T)\times \Gamma _u,\\ v_2&=0,&\text { on }&(0,T)\times \Gamma _u, \\ v(0)&=u_0,&\text { on }&\mathcal {O}. \end{aligned} \right. \end{aligned}$$

This first equation in the above system has the form

$$\begin{aligned} \partial _{t}v+v\cdot \nabla v+\nabla \pi =\nu \Delta v-L\left( v,w\right) \end{aligned}$$

with the affine function

$$\begin{aligned} L\left( v,w\right) =v\cdot \nabla w+w\cdot \nabla v+w\cdot \nabla w. \end{aligned}$$

For each $\omega \in \Omega$ fixed, the Navier–Stokes structure is preserved and the auxiliary equation for *v* with homogeneous boundary conditions is solvable similarly to the classical Navier–Stokes equations. Therefore, let us introduce the notion of weak solution of the deterministic problem (1.4) with random coefficients. Recall that *A* and *b* are (minus) the Stokes operator on $\mathbb {L}^2$ and defined in (2.16), respectively.

#### Definition 3.2

Given $u_0\in H$ and $w\in L^{4}\left( 0,T;\mathbb {L}^{4}\right)$, we say that

$$\begin{aligned} v\in C\left( \left[ 0,T\right] ;H\right) \cap L^{2}\left( 0,T;V\right) \end{aligned}$$

is a weak solution of Eq. (1.4) if

$$\begin{aligned}{} & {} \left\langle v\left( t\right) ,\phi \right\rangle -\int _{0}^{t}b\left( v\left( s\right) +w\left( s\right) ,\phi ,v\left( s\right) +w\left( s\right) \right) \,\textrm{d}s\\{} & {} \quad =\left\langle u_0,\phi \right\rangle -\int _{0}^{t}\left\langle v\left( s\right) ,A\phi \right\rangle \,\textrm{d}s \end{aligned}$$

for every $\phi \in \textsf{D}\left( A\right)$ and $t\in [0,T]$.

#### Theorem 3.3

For every $u_0\in H$ and $w\in L^{4}\left( 0,T;\mathbb {L} ^{4}\right)$, there exists a unique weak solution *v* of Eq. (1.4). Moreover, *v* satisfies for all $t\in [0,T]$

3.2 $$\begin{aligned}&\Vert v\left( t\right) \Vert ^{2}+2\int _{0} ^{t}\Vert \nabla v\left( s\right) \Vert _{L^{2}}^{2}\,\textrm{d}s=\Vert u_0\Vert ^{2} +2\int _{0}^{t}\left( b\left( v,v,w\right) +b\left( w,v,w\right) \right) \left( s\right) \,\textrm{d}s. \end{aligned}$$

If $\left( u_{0}^{n}\right) _{n\in \mathbb {N}}$ is a sequence in *H* converging to $u_{0}\in H$ and $\left( w^{n}\right) _{n\in \mathbb {N}}$ is a sequence in $L^{4}\left( 0,T;\mathbb {L} ^{4}\right)$ converging to $w\in L^{4}\left( 0,T;\mathbb {L} ^{4}\right)$, then the corresponding unique solutions $\left( v^{n}\right) _{n\in \mathbb {N}}$ converge to the corresponding solution *v* in $C\left( \left[ 0,T\right] ;H\right)$ and in $L^{2}\left( 0,T;V\right)$.

#### Proof

We split the proof into several steps.

*Step 1: Uniqueness*. Let $v^{\left( i\right) }$ be two solutions. The function $z=v^{\left( 1\right) }-v^{\left( 2\right) }$ satisfies

$$\begin{aligned}&\left\langle z\left( t\right) ,\phi \right\rangle -\int _{0}^{t}\left( b\left( v^{\left( 1\right) }+w,\phi ,v^{\left( 1\right) }+w\right) -b\left( v^{\left( 2\right) }+w,\phi ,v^{\left( 2\right) }+w\right) \right) \,\textrm{d}s\\&=-\int _{0}^{t}\left\langle z\left( s\right) ,A\phi \right\rangle \,\textrm{d}s \end{aligned}$$

for every $\phi \in \textsf{D}\left( A\right)$. A simple manipulation gives us

$$\begin{aligned}&b\left( v^{\left( 1\right) }+w,\phi ,v^{\left( 1\right) }+w\right) -b\left( v^{\left( 2\right) }+w,\phi ,v^{\left( 2\right) }+w\right) -b\left( z,\phi ,z\right) \\&\quad =b\left( v^{\left( 2\right) }+w,\phi ,z\right) +b\left( z,\phi ,v^{\left( 2\right) }+w\right) \end{aligned}$$

hence

$$\begin{aligned}&\left\langle z\left( t\right) ,\phi \right\rangle -\int _{0}^{t}b\left( z\left( s\right) ,\phi ,z\left( s\right) \right) \,\textrm{d}s=-\int _{0}^{t}\left\langle z\left( s\right) ,A\phi \right\rangle \,\textrm{d}s+\int _{0}^{t}\left\langle \widetilde{f}\left( s\right) ,\phi \right\rangle \,\textrm{d}s \end{aligned}$$

where

$$\begin{aligned} \widetilde{f}=-B\left( v^{\left( 2\right) }+w,z\right) -B\left( z,v^{\left( 2\right) }+w\right) \hspace{-0.1cm}. \end{aligned}$$

By Lemma 2.5, $\widetilde{f}\in L^{2}\left( 0,T;V^*\right)$. Then, by Theorem 2.6,

$$\begin{aligned} \Vert z\left( t\right) \Vert ^{2}+2\int _{0} ^{t}\Vert \nabla z\left( s\right) \Vert _{L^{2}}^{2}\,\textrm{d}s=2\int _{0}^{t}b\left( z,z,v^{\left( 2\right) }+w\right) \left( s\right) \,\textrm{d}s. \end{aligned}$$

Again by Lemma 2.5, we have

$$\begin{aligned} \left|b\left( z,z, v^{\left( 2\right) }+w\right) \right|&\le \left|b\left( z,z, v^{\left( 2\right) }\right) \right|+\left|b\left( z,z,w\right) \right|\\&\le \varepsilon \Vert z\Vert _{V}^{2}+\varepsilon \Vert z\Vert _{V}^{2}+\frac{C}{\varepsilon ^{3}}\Vert z\Vert ^{2}\Vert v^{\left( 2\right) }\Vert _{\mathbb {L}^4}^{4}\\&\quad +\varepsilon \Vert z\Vert _{V}^{2}+\varepsilon \Vert z\Vert _{V}^{2}+\frac{C}{\varepsilon ^{3}}\Vert z\Vert ^{2}\Vert w\Vert _{\mathbb {L}^4}^{4}\\&= 4\varepsilon \Vert z\Vert _{V}^{2}+\frac{C}{\varepsilon ^{3} }\Vert z\Vert ^{2}\left( \Vert v^{\left( 2\right) }\Vert _{\mathbb {L}^4}^{4}+\Vert w\Vert _{\mathbb {L}^4}^{4}\right) \hspace{-0.1cm}. \end{aligned}$$

Summarizing, with $4\varepsilon =1$, using the fact that $\Vert z\Vert _{V}^{2}=\Vert \nabla z\Vert _{L^{2}}^{2}$, renaming the constant *C*,

$$\begin{aligned} \Vert z\left( t\right) \Vert ^{2}+\int _{0}^{t}\Vert \nabla z\left( s\right) \Vert _{L^{2}}^{2}\,\textrm{d}s \le C\int _{0}^{t}\Vert z\left( s\right) \Vert ^{2}\left( 1+\Vert v^{\left( 2\right) }\left( s\right) \Vert _{\mathbb {L}^4}^{4}+\Vert w\left( s\right) \Vert _{\mathbb {L}^4}^{4}\right) \,\textrm{d}s. \end{aligned}$$

We conclude $z=0$ by the Gronwall lemma, using the assumption on *w* and the integrability properties of $v^{\left( 2\right) }$.

*Step 2: Existence*. Define the sequence $\left( v^{n}\right)$ by setting $v^{0}=0$ and for every $n\ge 0$, given $v^{n}\in C\left( \left[ 0,T\right] ;H\right) \cap L^{2}\left( 0,T;V\right)$, let $v^{n+1}$ be the solution of equation (2.15) with initial condition $u_0$ and with

$$\begin{aligned} -B\left( v^{n},w\right) -B\left( w,v^{n}\right) -B\left( w,w\right) \end{aligned}$$

in place of *f*. In particular

$$\begin{aligned}&\left\langle v^{n+1}\left( t\right) ,\phi \right\rangle -\int _{0} ^{t}b\left( v^{n+1}\left( s\right) ,\phi ,v^{n+1}\left( s\right) \right) \,\textrm{d}s\\&\quad =\left\langle u_0,\phi \right\rangle -\int _{0}^{t}\left\langle v^{n+1}\left( s\right) ,A\phi \right\rangle \,\textrm{d}s\\&\qquad -\int _{0}^{t}\left\langle \left( B\left( v^{n},w\right) +B\left( w,v^{n}\right) +B\left( w,w\right) \right) \left( s\right) ,\phi \right\rangle \,\textrm{d}s \end{aligned}$$

for every $\phi \in \textsf{D}\left( A\right)$. In order to claim that this definition is well done, we notice that

$$\begin{aligned} B\left( v^{n},w\right) ,B\left( w,v^{n}\right) ,B\left( w,w\right) \in L^{2}\left( 0,T;V^*\right) \end{aligned}$$

by Lemma 2.5.

Then let us investigate the convergence of $\left( v^{n}\right)$. First, let us prove a bound. From the previous identity and Theorem 2.6 we get

$$\begin{aligned}&\Vert v^{n+1}\left( t\right) \Vert ^{2}+2\int _{0}^{t}\Vert \nabla v^{n+1}\left( s\right) \Vert _{L^{2}} ^{2}\,\textrm{d}s\\&\quad =\Vert u_0\Vert ^{2} +2\int _{0}^{t}\left( b\left( v^{n},v^{n+1},w\right) +b\left( w,v^{n+1},v^{n}\right) +b\left( w,v^{n+1},w\right) \right) \left( s\right) \,\textrm{d}s. \end{aligned}$$

It gives us (using Lemma 2.5)

$$\begin{aligned}&\Vert v^{n+1}\left( t\right) \Vert ^{2}+\int _{0}^{t}\Vert \nabla v^{n+1}\left( s\right) \Vert _{L^{2}} ^{2}\,\textrm{d}s\\&\quad =\Vert u_0\Vert ^{2}+\varepsilon \int _{0}^{t}\Vert v^{n}\left( s\right) \Vert _{V}^{2}\,\textrm{d}s\\&\qquad +C_{\varepsilon }\int _{0}^{t}\Vert v^{n}\left( s\right) \Vert ^{2}\left( 1+\Vert w\left( s\right) \Vert _{\mathbb {L}^4} ^{4}\right) \,\textrm{d}s+C_{\varepsilon }\int _{0}^{t}\Vert w\left( s\right) \Vert _{\mathbb {L}^4}^{4}\,\textrm{d}s. \end{aligned}$$

Choosing a small constant $\varepsilon$, one can find $R>\Vert u_0\Vert ^{2}$ and $\overline{T}$ small enough, depending only from $\Vert u_0\Vert$ and $\Vert w\Vert _{L^4(0,T;\mathbb {L}^4)}$, such that if

3.3 $$\begin{aligned} \sup _{t\in \left[ 0,\overline{T}\right] }\Vert v^{n}\left( t\right) \Vert ^{2}\le R,\qquad \int _{0}^{\overline{T}}\Vert v^{n}\left( s\right) \Vert _{V}^{2}\,\textrm{d}s\le R \end{aligned}$$

then the same inequalities hold for $v^{n+1}$.

Set $w_{n}=v^{n}-v^{n-1}$, for $n\ge 1$. From the identity above,

$$\begin{aligned}&\left\langle w_{n+1}\left( t\right) ,\phi \right\rangle -\int _{0} ^{t}\left( b\left( v^{n+1},\phi ,v^{n+1}\right) -b\left( v^{n},\phi ,v^{n}\right) \right) \left( s\right) \,\textrm{d}s\\&\quad = -\int _{0}^{t}\left\langle w_{n+1}\left( s\right) ,A\phi \right\rangle \,\textrm{d}s-\int _{0}^{t}\left\langle \left( B\left( v^{n},w\right) -B\left( v^{n-1},w\right) \right) \left( s\right) ,\phi \right\rangle \,\textrm{d}s\\&\qquad -\int _{0}^{t}\left\langle \left( B\left( w,v^{n}\right) -B\left( w,v^{n-1}\right) \right) \left( s\right) ,\phi \right\rangle \,\textrm{d}s. \end{aligned}$$

Again as above, since

$$\begin{aligned}&b\left( v^{n+1},\phi ,v^{n+1}\right) -b\left( v^{n},\phi ,v^{n}\right) -b\left( w_{n+1},\phi ,w_{n+1}\right) \\&\quad =b\left( v^{n},\phi ,w_{n+1}\right) +b\left( w_{n+1},\phi ,v^{n}\right) \end{aligned}$$

we may rewrite it as

$$\begin{aligned}{} & {} \left\langle w_{n+1} \left( t\right) ,\phi \right\rangle -\int _{0} ^{t}b\left( w_{n+1}\left( s\right) ,\phi ,w_{n+1}\left( s\right) \right) \,\textrm{d}s\\{} & {} \quad =-\int _{0}^{t}\left\langle w_{n+1}\left( s\right) ,A\phi \right\rangle \,\textrm{d}s-\int _{0}^{t}\left\langle \left( B\left( w_{n},w\right) +B\left( w,w_{n}\right) \right) \left( s\right) ,\phi \right\rangle \,\textrm{d}s\\{} & {} \qquad +\int _{0}^{t}\left( b\left( v^{n},\phi ,w_{n+1}\right) +b\left( w_{n+1},\phi ,v^{n}\right) \right) \left( s\right) \,\textrm{d}s. \end{aligned}$$

One can check as above the applicability of Theorem 2.6 and get

$$\begin{aligned}{} & {} \Vert w_{n+1}\left( t\right) \Vert ^{2}+2\int _{0}^{t}\Vert \nabla w_{n+1}\left( s\right) \Vert _{L^{2}} ^{2}\,\textrm{d}s\\{} & {} \quad =2\int _{0}^{t}\left( b\left( w_{n},w_{n+1},w\right) +b\left( w,w_{n+1},w_{n}\right) \right) \left( s\right) \,\textrm{d}s\\{} & {} \qquad +2\int _{0}^{t}b\left( w_{n+1},w_{n+1},v^{n}\right) \left( s\right) \,\textrm{d}s. \end{aligned}$$

As above we deduce

$$\begin{aligned} |b\left( w_{n+1},w_{n+1},v^{n}\right) |\le \frac{1}{2}\Vert w_{n+1} \Vert _{V}^{2}+C\Vert w_{n+1}\Vert ^{2}\Vert v^{n}\Vert _{\mathbb {L}^4}^{4}. \end{aligned}$$

But

$$\begin{aligned} |b\left( w_{n},w_{n+1},w\right) +b\left( w,w_{n+1},w_{n}\right) |\le \frac{1}{2}\Vert w_{n+1}\Vert _{V}^{2}+\frac{1 }{4}\Vert w_{n}\Vert _{V}^{2}+C\Vert w_{n}\Vert ^{2}\Vert w\Vert _{\mathbb {L}^4}^{4}. \end{aligned}$$

Hence

$$\begin{aligned}&\Vert w_{n+1}\left( t\right) \Vert ^{2}+\int _{0}^{t}\Vert \nabla w_{n+1}\left( s\right) \Vert _{L^{2}} ^{2}\,\textrm{d}s\\&\quad \le C\int _{0}^{t}\Vert w_{n+1}\left( s\right) \Vert ^{2}\left( 1+\Vert v^{n}\left( s\right) \Vert _{\mathbb {L}^4} ^{4}\right) \,\textrm{d}s\\&\qquad +\frac{1}{4}\int _{0}^{t}\Vert w_{n}\left( s\right) \Vert _{V} ^{2}\,\textrm{d}s+C\int _{0}^{t}\Vert w_{n}\left( s\right) \Vert ^{2}\Vert w\left( s\right) \Vert _{\mathbb {L}^4}^{4}\,\textrm{d}s. \end{aligned}$$

Now we work under the bounds (3.3) and deduce, using the Gronwall lemma, for $\overline{T}$, depending only from $\Vert u_0\Vert$ and $\Vert w\Vert _{L^4(0,T;\mathbb {L}^4)}$, possibly smaller than the previous one,

$$\begin{aligned}&\sup _{t\in \left[ 0,\overline{T}\right] }\Vert w_{n+1}\left( t\right) \Vert ^{2}+\int _{0}^{\overline{T}}\Vert w_{n+1}\left( s\right) \Vert _{V}^{2}\,\textrm{d}s\\&\quad \le \frac{1}{2}\left( \sup _{t\in \left[ 0,\overline{T}\right] }\Vert w_{n}\left( t\right) \Vert ^{2}+\int _{0}^{\overline{T}}\Vert w_{n}\left( s\right) \Vert _{V}^{2}\,\textrm{d}s\right) \hspace{-0.1cm}. \end{aligned}$$

It implies that the sequence $\left( v^{n}\right)$ is Cauchy in $C\left( \left[ 0,\overline{T}\right] ;H\right) \cap L^{2}\left( 0,\overline{T};V\right)$. The limit *v* has the right regularity to be a weak solution and satisfies the weak formulation; in the identity above for $v^{n+1}$ and $v^{n}$ we may prove that

$$\begin{aligned} \int _{0}^{t}b\left( v^{n+1}\left( s\right) ,\phi ,v^{n+1}\left( s\right) \right) \,\textrm{d}s\rightarrow & {} \int _{0}^{t}b\left( v\left( s\right) ,\phi ,v\left( s\right) \right) \,\textrm{d}s\\ \int _{0}^{t}b\left( v^{n}\left( s\right) ,\phi ,w\left( s\right) \right) \,\textrm{d}s\rightarrow & {} \int _{0}^{t}b\left( v\left( s\right) ,\phi ,w\left( s\right) \right) \,\textrm{d}s\\ \int _{0}^{t}b\left( w\left( s\right) ,\phi ,v^{n}\left( s\right) \right) \,\textrm{d}s\rightarrow & {} \int _{0}^{t}b\left( w\left( s\right) ,\phi ,v\left( s\right) \right) \,\textrm{d}s. \end{aligned}$$

All these convergences can be proved easily by recalling the definition of *b*. Similarly, we can pass to the limit in the energy identity. After proving existence and uniqueness in $[0,\overline{T}]$ we can reiterate the existence procedure and in a finite number of steps cover the interval [0, *T*].

*Step 3: Continuous dependence on the data*. Let $v^n$ (resp. *v*) the unique solution of (1.4) with data $u_0^n,\ w^n$ (resp. $u_0,\ w$). Since $u_0^n\rightarrow u_0$ in *H* (resp. $w^n\rightarrow w$ in $L^4(0,T;\mathbb {L}^4)$) the family $(u_0^n)_{n\in \mathbb {N}}$ is bounded in *H* (resp. the family $(w^n)_{n\in \mathbb {N}}$ is bounded in $L^4(0,T;\mathbb {L}^4)$), by (3.2) one can show easily that the family $(v^n)_{n\in \mathbb {N}}$ is bounded in $C([0,T];H)\cap L^2(0,T;V)\hookrightarrow L^4(0,T;\mathbb {L}^4).$ Moreover for each $t\in [0,T]$, $z^n:=v^n-v$ satisfies the energy relation

3.4 $$\begin{aligned} \frac{1}{2}\Vert z^n(t)\Vert ^2+\int _0^t \Vert \nabla z^n(s)\Vert _{L^2}^2 \,\textrm{d}s&= \frac{1}{2}\Vert u_0^n-u_0\Vert ^2 \nonumber \\ {}&+\int _0^t b(v^n(s)+w^n(s),z^n(s),w^n(s)-w(s))\,\textrm{d}s \nonumber \\ {}&+\int _0^t b(z^n(s),z^n(s),v(s)+w(s))\,\textrm{d}s\nonumber \\ {}&+\int _0^t b(w^n(s)-w(s),z^n(s),v(s)+w(s))\,\textrm{d}s. \end{aligned}$$

We can easily bound the right hand side of relation (3.4) by Young’s inequality and Hölder’s inequality obtaining

3.5 $$\begin{aligned} \frac{1}{2}\Vert z^n(t)\Vert ^2&+\frac{1}{2}\int _0^t \Vert \nabla z^n(s)\Vert _{L^2}^2 \,\textrm{d}s\le \frac{1}{2}\Vert u_0^n-u_0\Vert ^2\nonumber \\ {}&+C\int _0^t \Vert z^n(s)\Vert ^2 \left( \Vert v(s)\Vert _{\mathbb {L}^4}^4+\Vert w(s)\Vert _{\mathbb {L}^4}^4\right) \,\textrm{d}s\nonumber \\ {}&+C\Vert w^n-w\Vert _{L^4(0,T;\mathbb {L}^4)}^2\left( \Vert w^n\Vert _{L^4(0,T;\mathbb {L}^4)}^2+\Vert w\Vert _{L^4(0,T;\mathbb {L}^4)}^2\right) \nonumber \\ {}&+C\Vert w^n-w\Vert _{L^4(0,T;\mathbb {L}^4)}^2\left( \Vert v^n\Vert _{L^4(0,T;\mathbb {L}^4)}^2+\Vert v\Vert _{L^4(0,T;\mathbb {L}^4)}^2\right) . \end{aligned}$$

Applying Gronwall’s inequality to relation (3.5) the claim follows immediately. $\square$

#### Remark 3.4

Freezing the variable $\omega \in \Omega$ and solving (1.4) for each $\omega$ does not allow to obtain information about the measurability properties of *v*. However, measurability of *v* with respect of the progressive $\sigma$-algebra follows from the continuity of the solution map with respect to $u_0$ and *w*. Therefore we have the required measurability properties for *v* with *w* being the mild solution of (1.3). In particular *v* has $\textbf{P}$-a.s. paths in $C(0,T;H)\cap L^2(0,T;V)$, it is progressively measurable with respect to these topologies and

3.6 $$\begin{aligned}&\left\langle v\left( t\right) ,\phi \right\rangle -\int _{0}^{t}b\left( v\left( s\right) +w\left( s\right) ,\phi ,v\left( s\right) +w\left( s\right) \right) \,\textrm{d}s \nonumber \\&\quad =\left\langle u_0,\phi \right\rangle -\int _{0}^{t}\left\langle v\left( s\right) ,A\phi \right\rangle \,\textrm{d}s \quad \textbf{P}-a.s. \end{aligned}$$

for every $\phi \in \textsf{D}\left( A\right)$ and $t\in [0,T]$.

#### Proof of Theorem 1.4

It follows immediately combining Proposition 3.1, Theorem 3.3 and Remark 3.4.

## Interior regularity

### Stokes equations

As in the proof of Theorem 1.4, by a stopping time argument we may assume that $h_b$ is also $L^p(\Omega )$-integrable, cf. the beginning of the proof of Proposition 3.1. This fact will be used below without further mentioning it. We start showing a lemma, concerning the relation between the mild and the weak formulation of (1.3) defined below.

#### Definition 4.1

Let Hypothesis 1.1 be satisfied. A stochastic process *w* is a weak solution of (1.3) if it is $\mathcal {F}$-progressively measurable with $\textbf{P}-a.s.$ paths in

$$\begin{aligned} w \in C([0,T];H)\cap L^4(0,T;\mathbb {L}^4) \end{aligned}$$

and for each $\phi \in \textsf{D}(A)$

4.1 $$\begin{aligned} \langle w(t),\phi \rangle =&-\int _{0}^{t}\langle w(s),A\phi \rangle \ \,\textrm{d}s+\int _0^t \langle h_b(s),\phi \rangle _{W^{-\alpha ,q}(\Gamma _u),W^{\alpha ,q'}(\Gamma _u)} \,\textrm{d}W_{\mathcal {H}}(s) \end{aligned}$$

for each $$t \in [0,T],\ \textbf{P}-a.s.$$

Note that the last term in (4.1) is well-defined as $\alpha <1/2$ and $q'< 2$.

#### Remark 4.2

In the definition above, the term $\langle h_b(s),\phi \rangle _{W^{-\alpha ,q}(\Gamma _u),W^{\alpha ,q'}(\Gamma _u)}$ is given by the following linear operator on $\mathcal {H}$:

$$\begin{aligned} \mathcal {H}\ni h'\mapsto \langle h_b(s)h',\phi \rangle _{W^{-\alpha ,q}(\Gamma _u),W^{\alpha ,q'}(\Gamma _u)}=L_{\phi } (h_b(s)h') \end{aligned}$$

where $L_{\phi }:=\langle \cdot ,\phi \rangle _{W^{-\alpha ,q}(\Gamma _u),W^{\alpha ,q'}(\Gamma _u)}$. By the ideal property of $\gamma$-spaces and $\gamma (\mathcal {H}, W^{-\alpha ,q}(\Gamma _u))=W^{-\alpha ,q}(\Gamma _u;\mathcal {H})$ we have

$$\begin{aligned} \Vert \langle h_b(s),\phi \rangle _{W^{-\alpha ,q}(\Gamma _u),W^{\alpha ,q'}(\Gamma _u)}\Vert _{\mathcal {H}^*}\lesssim \Vert h_b(s)\Vert _{W^{-\alpha ,q}(\Gamma _u;\mathcal {H})}\Vert \phi \Vert _{W^{\alpha ,q'}(\Gamma _u)}, \end{aligned}$$

a.e. on $(0,T)\times \Omega$. Whence, the stochastic integral in (4.1) is well-defined with scalar value as

$$\begin{aligned}&\textbf{E}\left[ \Big | \int _0^T \langle h_b(s),\phi \rangle _{W^{-\alpha ,q}(\Gamma _u),W^{\alpha ,q'}(\Gamma _u)} \,\textrm{d}W_{\mathcal {H}}(s)\Big |^2\right] \\&\quad = \textbf{E} \left[ \int _0^T \Vert \langle h_b(s),\phi \rangle _{W^{-\alpha ,q}(\Gamma _u),W^{\alpha ,q'}(\Gamma _u)}\Vert _{\mathcal {H}^*}^2 \,\textrm{d}s\right] \\&\quad \lesssim \textbf{E} \left[ \int _0^T \Vert h_b(s)\Vert _{W^{-\alpha ,q}(\Gamma _u;\mathcal {H})}^2\Vert \phi \Vert _{W^{-\alpha ,q'}(\Gamma _u)}^2 \,\textrm{d}s\right] <\infty , \end{aligned}$$

where the last estimate follows from Hypothesis 1.1.

#### Lemma 4.3

Let Hypothesis 1.1 be satisfied. There exists a unique weak solution of (1.3) in the sense of Definition 4.1 and it is given by the formula (3.1).

#### Proof

We split the proof into two steps.

*Step 1: There exists a unique weak solution of* (1.3) *and it is necessarily given by the mild formula* (3.1). Let $\psi \in C^1([0,T];\textsf{D}(A))$. Arguing as in the first step of the proof of [27, Theorem 1.7], see also [26, Proposition 17], one can readily check that *w* satisfies

4.2 $$\begin{aligned} \langle w(t),\psi (t)\rangle =&\int _0^t\langle w(s),\partial _s\psi (s)\rangle \,\textrm{d}s-\int _{0}^{t}\langle w(s),A\psi (s)\rangle \,\textrm{d}s\nonumber \\ {}&+\int _0^t \langle h_b(s),\psi (s)\rangle _{W^{-\alpha ,q}(\Gamma _u),W^{\alpha ,q'}(\Gamma _u)} \,\textrm{d}W_{\mathcal {H}}(s) \end{aligned}$$

for each $t \in [0,T],\ \textbf{P}-a.s.$ The stochastic integral in the relation above is well-defined arguing as in Remark 4.2. Now consider $\phi \in \textsf{D}(A^2)$ and use $\psi _t(s)=S_{q'}(t-s)\phi ,\ s\in [0,t]$ as test function in (4.2) obtaining, since $S_{q'}(t)|_{H}=S(t)$,

4.3 $$\begin{aligned} \langle w(t),\phi \rangle =&\int _0^t \langle h_b(s),S_{q'}(t-s)\phi \rangle _{W^{-\alpha ,q}(\Gamma _u),W^{\alpha ,q'}(\Gamma _u)} \,\textrm{d}W_{\mathcal {H}}(s). \end{aligned}$$

Recalling the definition of the Neumann map $\mathcal {N}$, (4.3) can be rewritten as

4.4 $$\begin{aligned} \langle w(t),\phi \rangle =&\int _0^t \langle \mathcal {N}[h_b(s)],A_{q'}S_{q'}(t-s)\phi \rangle \,\textrm{d}W_{\mathcal {H}}(s). \end{aligned}$$

Then, exploiting the self-adjointness property of $S_q$ and $A_q$ we have that weak solutions of (1.3) satisfy the mild formulation. Therefore they are unique.

*Step 2: The mild formula* (3.1) *is a weak solution of* (1.3) *in the sense of* Definition 4.1. We begin by noticing that *w* has the required regularity due to Proposition 3.1. Let us test our mild formulation (3.1) against functions $\phi \in \textsf{D}(A^2)\subseteq \textsf{D}(A_{q'}^2)$. It holds, since $S_{q'}(t)|_{H}=S(t),\ A_{q'}|_{\textsf{D}(A)}=A$ and exploiting self-adjointness property of $S_q$ and $A_q$

$$\begin{aligned} \langle w(t),\phi \rangle&= \int _0^t \langle \mathcal {N}[h_b(s)],AS(t-s)\phi \rangle \,\textrm{d}W_{\mathcal {H}}(s)\\ {}&=\int _0^t \langle h_b(s),S(t-s)\phi \rangle _{W^{-\alpha ,q}(\Gamma _u),W^{\alpha ,q'}(\Gamma _u)} \,\textrm{d}W_{\mathcal {H}}(s)\quad \textbf{P}-a.s., \end{aligned}$$

where in the last step we used the definition of Neumann map. In order to complete the proof of this step it is enough to show that

4.5 $$\begin{aligned} \int _0^t \langle h_b(s),S(t-s)\phi \rangle _{W^{-\alpha ,q}(\Gamma _u),W^{\alpha ,q'}(\Gamma _u)} \,\textrm{d}W_{\mathcal {H}}(s)=-\int _0^t \langle w(s),A\phi \rangle \,\textrm{d}s\nonumber&\\ +\int _0^t \langle h_b(s),\phi \rangle _{W^{-\alpha ,q}(\Gamma _u),W^{\alpha ,q'}(\Gamma _u)} \,\textrm{d}W_{\mathcal {H}}(s) \quad \textbf{P}-a.s.&\end{aligned}$$

Relation (4.5) is true. Indeed,

4.6 $$\begin{aligned} \int _0^t \langle w(s),A\phi \rangle \,\textrm{d}s&=\int _0^t \,\textrm{d}s \int _0^s \langle \mathcal {N}[h_b(r)],S(s-r)A^2 \phi \rangle \,\textrm{d}W_{\mathcal {H}}(r)\quad \textbf{P}-a.s. \end{aligned}$$

The double integrals in (4.6) can be exchanged via stochastic Fubini’s Theorem, see [20], since

$$\begin{aligned}&\int _0^t \,\textrm{d}s\left( \textbf{E}\left[ \int _0^s \,\textrm{d}r \Vert \langle \mathcal {N}[h_b(r)],S(s-r)A^2 \phi \rangle \Vert _{\mathcal {H}}^2\right] \right) ^{1/2}\\ {}&\quad \le C(T,q)\Vert A^2 \phi \Vert _{\mathbb {L}^2}\textbf{E}\left[ \Vert h_b\Vert _{L^2(0,T;W^{-\alpha ,q}(\Gamma _u;\mathcal {H}))}^2\right] ^{1/2} <+\infty \end{aligned}$$

Therefore the double integral in the right hand side of (4.6) can be rewritten as

$$\begin{aligned}&\int _0^t \,\textrm{d}s \int _0^s \langle \mathcal {N}[h_b(r)],S(s-r)A^2 \phi \rangle \,\textrm{d}W_{\mathcal {H}}(r)\\&\quad =\int _0^t \,\textrm{d}W_{\mathcal {H}}(r) \int _r^t \langle \mathcal {N}[h_b(r)],S(s-r)A^2 \phi \rangle \,\textrm{d}s\\&\quad =\int _0^t \langle \mathcal {N}[h_b(r)],A \phi \rangle \,\textrm{d}W_{\mathcal {H}}(r)\\&\qquad -\int _0^t \langle \mathcal {N}[h_b(r)],AS(t-r)\phi \rangle \,\textrm{d}W_{\mathcal {H}}(r)\\&\quad = \int _0^t \langle h_b(r), \phi \rangle _{W^{-\alpha ,q}(\Gamma _u),W^{\alpha ,q'}(\Gamma _u)} \,\textrm{d}W_{\mathcal {H}}(r)\\&\qquad -\int _0^t \langle h_b(r),S(t-r)\phi \rangle _{W^{-\alpha ,q}(\Gamma _u),W^{\alpha ,q'}(\Gamma _u)} \,\textrm{d}W_{\mathcal {H}}(r)\quad \textbf{P}-a.s. \end{aligned}$$

Inserting this expression in (4.6), (4.5) holds and the proof is complete. $\square$

Thanks to the weak formulation guaranteed by Lemma 4.3 we can easily obtain the interior regularity of the linear stochastic problem (1.3). Let $N_0$ be the $\textbf{P}$ null measure set where at least one between $w\notin C([0,T];H)\cap L^4(0,T;\mathbb {L}^4)$, $v\notin C([0,T];H)\cap L^2(0,T;V)$, (4.1) and (3.6) is not satisfied. In the following we will work pathwise in $\Omega \smallsetminus N_0$ even if not specified.

#### Corollary 4.4

Let Hypothesis 1.1 be satisfied. Let *w* be the unique weak solution of (1.3) in the sense of Definition 4.1. Then, for all $0<t_1\le t_2<T,$ $x_0\in \mathcal {O}$, $r>0$ such that $\textrm{dist}({B(x_0,r)}, \partial \mathcal {O})>0$,

$$\begin{aligned} w\in C([t_1,t_2], C^{\infty }({B(x_0,r)};\mathbb {R}^2)) \quad \textbf{P}-a.s. \end{aligned}$$

#### Proof

Denote $\omega _w={\text {curl}}w\in C([0,T];H^{-1}(\mathcal {O}))\ \textbf{P}-a.s.$ Since $\textrm{dist}({B(x_0,r)}, \partial \mathcal {O})>0,\ 0<t_1\le t_2<T$ we can find $\varepsilon$ small enough such that $0<t_1-2\varepsilon<t_2+2\varepsilon <T,\ \textrm{dist}({B(x_0,r+2\varepsilon )}, \partial \mathcal {O})>0$. Let us consider $\psi \in C^{\infty }_c(\mathcal {O})$ and use $\nabla ^{\perp }\psi$ as test function in (4.1). This implies that $\omega _w$ is a distributional solution of the heat equation

$$\begin{aligned} \partial _t \omega _w=\Delta \omega _w. \end{aligned}$$

Since $\omega _w$ solves the heat equation in distributions, a standard localization argument and regularity results for the heat equation (see e.g. [49, Chapter 6, Section 1]) imply that

$$\begin{aligned} \omega _w\in C([t_1-\varepsilon ,t_2+\varepsilon ],C^{\infty }(B(x_0,r+\varepsilon )))\quad \textbf{P}-a.s. \end{aligned}$$

Let us now consider a test function $\phi \in C^{\infty }_c(B(x_0,r+\varepsilon ))$ identically equal to one on $B(x_0,r+\varepsilon /2)$. Since ${\text {div}} w=0$, we have that $\hat{w}=\phi w$ solves the elliptic problem

$$\begin{aligned} \Delta \hat{w}=\nabla ^{\perp }\omega _w\phi +\Delta \phi w+2\nabla \phi \cdot \nabla w,\quad \hat{w}|_{\partial B(x_0,r+\varepsilon )}=0. \end{aligned}$$

Since $w\in C([t_1-\varepsilon ,t_2+\varepsilon ];L^2(B(x_0,r+\varepsilon )))\ \textbf{P}-a.s.$ by Proposition 3.1, it follows that

$$\begin{aligned} \nabla ^{\perp }\omega _w\phi +\Delta \phi w+2\nabla \phi \cdot \nabla w\in C([t_0-\varepsilon ,T];H^{-1}(B(x_0,r+\varepsilon )))\quad \textbf{P}-a.s. \end{aligned}$$

Therefore, by standard elliptic regularity theory

$$\begin{aligned} \hat{w}\in C([t_1-\varepsilon ,t_2+\varepsilon ];H^{1}(B(x_0,r+\varepsilon )))\quad \textbf{P}-a.s. \end{aligned}$$

From the fact that $\phi \equiv 1$ on $B(x_0,r+\varepsilon /2)$ it follows that

$$\begin{aligned} w\in C([t_1-\varepsilon /4,t_2+\varepsilon /4];H^{1}(B(x_0,r+\varepsilon /4)))\quad \textbf{P}-a.s. \end{aligned}$$

Therefore, the required regularity of *w* is established by inductively reiterating this argument and by considering test functions $\phi \in C^{\infty }_c(B(x_0,r+\frac{\varepsilon }{2^{2n}}))$ identically equal to one on $B(x_0,r+\frac{\varepsilon }{2^{2n+1}})$. $\square$

### Auxiliary Navier–Stokes type equations

In order to deal with the interior regularity of (1.4) we perform a Serrin type argument, see [36, 47]. The regularity of *w* guaranteed by Corollary 4.4 will play a crucial role to treat the linear terms appearing in (1.4). We start with the following lemma.

#### Lemma 4.5

Let Hypothesis 1.1 be satisfied. Let *v* be the unique solution of (1.4) in the sense of Definition 3.2, where *w* is as in Corollary 4.4. Then, for all $0<t_1\le t_2<T,$ $x_0\in \mathcal {O}$, $r>0$ such that $\textrm{dist}({B(x_0,r)}, \partial \mathcal {O})>0$,

$$\begin{aligned} v\in C([t_1,t_2], H^{3/2}({B(x_0,r)};\mathbb {R}^2)) \quad \textbf{P}-a.s. \end{aligned}$$

#### Proof

As described in Lemma 4.3, arguing as in the proof of [27, Theorem 1.7], we can extend the weak formulation satisfied by *v* to time dependent test functions $\phi \in C^1([0,T]; H)\cap C([0,T];\textsf{D}(A))$ obtaining that for each $t\in [0,T]$

$$\begin{aligned} \langle v(t),\phi (t)\rangle -\langle u_0,\phi (0)\rangle&= \int _0^t \langle v(s),\partial _s \phi (s)\rangle \,\textrm{d}s- \int _{0}^{t}\left\langle v\left( s\right) ,A\phi (s)\right\rangle \,\textrm{d}s\\ {}&\quad +\int _{0}^{t}b\left( v\left( s\right) +w\left( s\right) ,\phi (s),v\left( s\right) +w\left( s\right) \right) \,\textrm{d}s \quad \textbf{P}-a.s. \end{aligned}$$

Choosing $\phi =-\nabla ^{\perp }\chi ,\ \chi \in C^{\infty }_c((0,T)\times \mathcal {O})$ in the weak formulation above and denoting by

$$\begin{aligned} \omega ={\text {curl}}v&\in C([0,T];H^{-1})\cap L^2((0,T)\times \mathcal {O})\quad \textbf{P}-a.s., \\ \omega _w={\text {curl}}w&\in C([t_1,t_2], C^{\infty }({B(x_0,r)}))\quad \textbf{P}-a.s. \end{aligned}$$

it follows that

$$\begin{aligned} -\int _0^T \langle \omega (s),\partial _s\chi (s)\rangle +\langle \omega (s),\Delta \chi (s)\rangle \,\textrm{d}s&=\int _0^T \langle {\text {curl}}(w(s)\otimes w(s)),\nabla \chi (s)\rangle \,\textrm{d}s \\ {}&+\int _0^T \langle {\text {curl}}(w(s)\otimes v(s)),\nabla \chi (s)\rangle \,\textrm{d}s\\ {}&+\int _0^T\langle {\text {curl}}(v(s)\otimes w(s)),\nabla \chi (s)\rangle \,\textrm{d}s\\ {}&+\int _0^T \langle \omega (s), v(s)\cdot \nabla \chi (s)\rangle \,\textrm{d}s. \end{aligned}$$

This means that $\omega$ is a distributional solution in $(0,T)\times \mathcal {O}$ of the partial differential equation

$$\begin{aligned} \partial _t \omega +v\cdot \nabla \omega&=\Delta \omega -{\text {div}}\bigg ({\text {curl}}(w(s)\otimes w(s))\\&\quad +{\text {curl}}(w(s)\otimes v(s))+{\text {curl}}(v(s)\otimes w(s))\bigg ). \end{aligned}$$

Since $\textrm{dist}({B(x_0,r)}, \partial \mathcal {O})>0,\ 0<t_1\le t_2<T$ we can find $\varepsilon$ small enough such that $0<t_1-2\varepsilon<t_1\le t_2<t_2+2\varepsilon <T,\ \textrm{dist}({B(x_0,r+2\varepsilon )}, \partial \mathcal {O})>0$. Let us consider $\psi \in C^{\infty }_c((0,T)\times \mathcal {O})$ supported in $[t_1-\varepsilon ,t_2+\varepsilon ]\times B(x_0,r+\varepsilon )$ such that it is equal to one in $[t_1-\varepsilon /2,t_2+\varepsilon /2]\times B(x_0,r+\varepsilon /2)$. Let us denote by $\tilde{\omega }=\omega \psi \in L^2((0,T)\times \mathbb {R}^2)$ supported in $[t_1-\varepsilon ,t_2+\varepsilon ]\times B(x_0,r+\varepsilon )$, then $\tilde{\omega }$ is a distributional solution in $(0,T)\times \mathbb {R}^2$ of

4.7 $$\begin{aligned} \partial _t \tilde{\omega }&= \Delta \tilde{\omega }-v\cdot \nabla \tilde{\omega }-w\cdot \nabla \tilde{\omega }+g \end{aligned}$$

with

$$\begin{aligned} g&=\partial _t\psi \omega -2\nabla \psi \cdot \nabla \omega -\Delta \psi \omega +v\cdot \nabla \psi \omega \\ {}&-\psi w\cdot \nabla \omega _w-\psi v\cdot \nabla \omega _w+w\cdot \nabla \psi \omega . \end{aligned}$$

Due to Corollary 4.4 the terms

$$\begin{aligned} -\psi w\cdot \nabla \omega _w-\psi v\cdot \nabla \omega _w+w\cdot \nabla \psi \omega \in L^2((0,T)\times \mathbb {R}^2)\quad \textbf{P}-a.s. \end{aligned}$$

Therefore $g\in L^2(0,T;H^{-1}(\mathbb {R}^2))+L^1(0,T;L^2(\mathbb {R}^2))\ \textbf{P}-a.s.$ Then, arguing as in the first step of the proof of [36, Theorem 13.2], the fact that $\tilde{\omega }$ is a distributional solution of (4.7) implies that $\tilde{\omega }\in C([0,T];L^2(\mathbb {R}^2))\cap L^2(0,T;H^1(\mathbb {R}^2)).$ Therefore

$$\begin{aligned} \omega \in&C([t_1-\varepsilon /4,t_2+\varepsilon /4];L^2(B(x_0,r+\varepsilon /4)))\\ {}&\cap L^2(t_1-\varepsilon /4,t_2+\varepsilon /4;H^1(B(x_0,r+\varepsilon /4)))\quad \textbf{P}-a.s. \end{aligned}$$

Introducing $\phi \in C^{\infty }_c(B(x_0,r+\varepsilon /4))$ equal to one in $B(x_0,r+\varepsilon /8)$, since $\omega ={\text {curl }}v$, then $\phi v$ satisfies

4.8 $$\begin{aligned} \Delta (\phi v)=\nabla ^{\perp }\omega \phi +\Delta \phi v+2\nabla \phi \cdot \nabla v ,\quad (\phi v)|_{\partial B(x_0,r+\varepsilon /4)}=0. \end{aligned}$$

From the regularity of $\omega$, by standard elliptic regularity theory (see for example [6]), it follows that $\phi v\in C([t_1-\varepsilon /4,t_2+\varepsilon /4];H^1(B(x_0,r+\varepsilon /4);\mathbb {R}^2))\cap L^2(t_1-\varepsilon /4,t_2+\varepsilon /4;H^2(B(x_0,r+\varepsilon /4);\mathbb {R}^2))\ \textbf{P}-a.s$. Therefore, since $\phi \equiv 1$ on $B(x_0,r+\varepsilon /8)$

4.9 $$\begin{aligned} v\in&C([t_1-\frac{\varepsilon }{16},t_2+\frac{\varepsilon }{16}];H^1(B(x_0,r+\frac{\varepsilon }{16});\mathbb {R}^2))\nonumber \\ {}&\cap L^2(t_1-\frac{\varepsilon }{16},t_2+\frac{\varepsilon }{16};H^2(B(x_0,r+\frac{\varepsilon }{16});\mathbb {R}^2))\quad \textbf{P}-a.s. \end{aligned}$$

Let us now consider $\hat{\psi }\in C^{\infty }_c((t_1-\frac{\varepsilon }{16},t_2+\frac{\varepsilon }{16})\times B(x_0,r+\frac{\varepsilon }{16}))$ such that it is equal to one in $[t_1-\frac{\varepsilon }{32},t_2+\frac{\varepsilon }{32}]\times B(x_0,r+\frac{\varepsilon }{32})$. Let us denote by $\hat{\omega }=\omega \hat{\psi }\in C([0,T];L^2(\mathbb {R}^2))\cap L^2(0,T; H^1(\mathbb {R}^2))$ supported in $(t_1-\frac{\varepsilon }{16},t_2+\frac{\varepsilon }{16})\times B(x_0,r+\frac{\varepsilon }{16})$, then $\hat{\omega }$ is a distributional solution in $(0,T)\times \mathbb {R}^2$ of

4.10 $$\begin{aligned} \partial _t \hat{\omega }&= \Delta \hat{\omega }+\hat{g} \end{aligned}$$

with

$$\begin{aligned} \hat{g}&=-v\cdot \nabla \hat{\omega }-w\cdot \nabla \hat{\omega }+\partial _t\hat{\psi } \omega -2\nabla \hat{\psi }\cdot \nabla \omega -\Delta \hat{\psi } \omega +v\cdot \nabla \hat{\psi } \omega \\ {}&-\hat{\psi } w\cdot \nabla \omega _w-\hat{\psi } v\cdot \nabla \omega _w+w\cdot \nabla \hat{\psi }\omega . \end{aligned}$$

By Corollary 4.4 and relation (4.9) it follows that $\hat{g}\in L^2(0,T;H^{-1/2}(\mathbb {R}^2))\ \textbf{P}-a.s.$ Therefore $\hat{\omega }\in C([0,T];H^{1/2}(\mathbb {R}^2))\cap L^2(0,T;H^{3/2}(\mathbb {R}^2)) \ \textbf{P}-a.s.$ and arguing as above

$$\begin{aligned} v\in&C([t_1-\frac{\varepsilon }{64},{t}_2+\frac{\varepsilon }{64}], H^{3/2}({B(x_0,{r}+\frac{\varepsilon }{64})};\mathbb {R}^2))\\ {}&\cap L^2({t}_1-\frac{\varepsilon }{64},{t}_2+\frac{\varepsilon }{64}, H^{5/2}({B(x_0,{r}+\frac{\varepsilon }{64})};\mathbb {R}^2))\quad \textbf{P}-a.s. \end{aligned}$$

This concludes the proof of Lemma 4.5. $\square$

#### Corollary 4.6

Let Hypothesis 1.1 be satisfied. Let *v* be the unique weak solution of (1.4) in the sense of Definition 3.2, where *w* is as in 4.4. Then, for all $0<t_1\le t_2<T,$ $x_0\in \mathcal {O}$, $r>0$ such that $\textrm{dist}({B(x_0,r)}, \partial \mathcal {O})>0$,

$$\begin{aligned} v\in C([t_1,t_2]; C^{\infty }({B(x_0,r)};\mathbb {R}^2))\quad \textbf{P}-a.s. \end{aligned}$$

#### Proof

Since $\textrm{dist}({B(x_0,r)}, \partial \mathcal {O})>0,\ 0<t_1\le t_2<T$ we can find $\varepsilon$ small enough such that $0<t_1-2\varepsilon<t_1\le t_2<t_2+2\varepsilon <T,\ \textrm{dist}({B(x_0,r+2\varepsilon )}, \partial \mathcal {O})>0$ and $\psi \in C^{\infty }_c((0,T)\times \mathcal {O})$ supported in $[t_1-\varepsilon ,t_2+\varepsilon ]\times B(x_0, r+\varepsilon )$ such that it is equal to one in $[t_1+\varepsilon /2,t_2+\varepsilon /2]\times B(x_0,r+\varepsilon /2)$. From Lemma 4.5 and Sobolev embedding theorem we know that $v\in C([t_1-\varepsilon ,t_2+\varepsilon ];L^{\infty }(B(x_0,{r}+\varepsilon );\mathbb {R}^2))\ \textbf{P}-a.s.$ Denoting by

$$\begin{aligned}&\omega ={\text {curl}}v\in C([0,T];H^{-1})\cap L^2((0,T)\times \mathcal {O}), \\ {}&\omega _w={\text {curl}}w\in C([t_1-2\varepsilon ,t_2+\varepsilon ], C^{\infty }({B(x_0,r+2\varepsilon )}))\quad \textbf{P}-a.s. \end{aligned}$$

and $\tilde{\omega }=\omega \psi \in L^2((0,T)\times \mathbb {R}^2)$ supported in $[t_1-\varepsilon ,t_2+\varepsilon ]\times B(x_0,r+\varepsilon )$, then, arguing as in the proof of Lemma 4.5, it follows that $\tilde{\omega }$ is a distributional solution in $(0,T)\times B(x_0, r+\varepsilon )$ of

4.11 $$\begin{aligned} \partial _t \tilde{\omega }&= \Delta \tilde{\omega }+\tilde{g} \end{aligned}$$

with

$$\begin{aligned} \tilde{g}&=-v\cdot \nabla \tilde{\omega }-w\cdot \nabla \tilde{\omega }+\partial _t{\psi } \omega -2\nabla {\psi }\cdot \nabla \omega -\Delta {\psi } \omega +v\cdot \nabla {\psi } \omega \\ {}&-{\psi } w\cdot \nabla \omega _w-{\psi } v\cdot \nabla \omega _w+w\cdot \nabla {\psi }\omega . \end{aligned}$$

From the regularity of $\omega ,\ v,\ \tilde{\omega },\ \omega _w,\ w$, then ${\tilde{g}} \in L^2(t_1-\varepsilon ,t_2+\varepsilon ;H^{-1}(B(x_0,r+\varepsilon )))\ \textbf{P}-a.s$. By standard regularity theory for the heat equation, see for example Step 2 in [36, Theorem 13.1], a solution of (4.11) with ${\tilde{g}} \in L^2(t_1-\varepsilon ,t_2+\varepsilon ;H^{k-1}(B(x_0,r+\varepsilon ))),\ k\in \mathbb {N}$, belongs to $C([{t}_1-\varepsilon /2,{t}_2+\varepsilon /2];H^k(B(x_0,{r}+\varepsilon /2)))\cap L^2({t}_1-\varepsilon /2,{t}_2+\varepsilon /2;H^{k+1}(B(x_0,{r}+\varepsilon /2)))$. Therefore

$$\begin{aligned} \tilde{\omega }&\in C([{t}_1-\varepsilon /2,{t}_2+\varepsilon /2];L^2(B(x_0,{r}+\varepsilon /2)))\\&\cap L^2({t}_1-\varepsilon /2,{t}_2+\varepsilon /2;H^{1}(B(x_0,{r}+\varepsilon /2)))\quad \textbf{P}-a.s. \end{aligned}$$

which implies

$$\begin{aligned} \omega&\in C([{t}_1-\varepsilon /4,{t}_2+\varepsilon /4];L^2(B(x_0,{r}+\varepsilon /4)))\\ {}&\cap L^2({t}_1-\varepsilon /4,{t}_2+\varepsilon /4;H^{1}(B(x_0,{r}+\varepsilon /4)))\quad \textbf{P}-a.s. \end{aligned}$$

since $\psi \equiv 1$ on $({t}_1-\varepsilon /2,{t}_2+\varepsilon /2)\times B(x_0,{r}+\varepsilon /2).$ Considering now $\phi \in C^{\infty }_c(\mathcal {O})$ supported on $B(x_0,r+\varepsilon /4)$ such that $\phi \equiv 1$ on $B(x_0,{r}+\varepsilon /8)$, since ${\text {curl}}v=\omega$ then $\phi v$ satisfies

4.12 $$\begin{aligned} \Delta (\phi v)=\nabla ^{\perp }\omega \phi +\Delta \phi v+2\nabla \phi \cdot \nabla v,\quad (\phi v)|_{\partial B(x_0,r+\varepsilon /4)}=0. \end{aligned}$$

Since

$$\begin{aligned}&\nabla ^{\perp }\omega \phi +\Delta \phi v+2\nabla \phi \cdot \nabla v \in C([{t}_1-\varepsilon /4,{t}_2+\varepsilon /4];H^{-1}(B(x_0,{r}+\varepsilon /4)))\\ {}&\cap L^2({t}_1-\varepsilon /4,{t}_2+\varepsilon /4;L^{2}(B(x_0,{r}+\varepsilon /4)))\quad \textbf{P}-a.s., \end{aligned}$$

by standard elliptic regularity theory (see for example [6]),

$$\begin{aligned} \phi v&\in C([{t}_1-\varepsilon /4,{t}_2+\varepsilon /4];H^1(B(x_0,{r}+\varepsilon /4)))\\ {}&\cap L^2({t}_1-\varepsilon /4,{t}_2+\varepsilon /4;H^{2}(B(x_0,{r}+\varepsilon /4)))\quad \textbf{P}-a.s. \end{aligned}$$

Since $\phi \equiv 1$ on $B(x_0,{r}+\varepsilon /8)$ then

$$\begin{aligned} v&\in C([{t}_1-\frac{\varepsilon }{16},{t}_2+\frac{\varepsilon }{16}];H^{1}(B(x_0,r+\frac{\varepsilon }{16})))\\ {}&\cap L^2({t}_1-\frac{\varepsilon }{16},{t}_2+\frac{\varepsilon }{16};H^2(B(x_0,{r}+\frac{\varepsilon }{16})))\quad \textbf{P}-a.s. \end{aligned}$$

Reiterating the argument as in Step 3 in [36, Theorem13.1] the thesis follows. $\square$

#### Proof of Theorem 1.7

The claim follows by Corollaries 4.4 and 4.6 and a localization argument. Moreover, to obtain the claimed smoothness up to time $t=T$, let us consider the extension by 0 of $h_b$ on $[0,T+1]$, i.e.

$$\begin{aligned} \widetilde{h}_b(t)= \left\{ \begin{aligned}&h_b(t),\quad&\text {if }\ {}&t\in (0,T),\\&0,&\text {if }\ {}&t\in (T,T+1). \end{aligned}\right. \end{aligned}$$

Let $\widetilde{u}$ be the unique weak solution (1.2) provided by Theorem 1.4 with *T* replaced by $T+1$. Then, by Corollary 4.4, Corollary 4.6 and a standard covering argument, for all $t_0\in (0,T)$, $\mathcal {O}_0\subset \mathcal {O}$ such that $\textrm{dist}({\mathcal {O}_0}, \partial \mathcal {O})>0$,

4.13 $$\begin{aligned} \widetilde{u}\in C([t_0,T];C^{\infty }(\mathcal {O}_0;\mathbb {R}^2))\quad \textbf{P}-a.s. \end{aligned}$$

Now, let *u* be the unique weak solution of (1.2) provided by Theorem 1.4. By uniqueness, we have $u=\widetilde{u}|_{[0,T]}$ and the conclusion follows from (4.13).

## Data Availability

Data sharing not applicable to this article as no data sets were generated or analyzed during the current study.

## Appendix A: $H^{\infty }$-calculus for the Stokes operator

In this appendix we prove Theorem 2.1. Here we use the transference result proven in [42]. In this section we also need the concept of $\mathcal {R}$-sectoriality, which can be again found in [43, Chapters 3 and 4] and [32, Chapter 10].

To discuss the main idea in the proof of Theorem 2.1, let us define the operator $B_q v:=- \Delta v$ on $L^q(\mathcal {O};\mathbb {R}^2)$ with domain

$$\begin{aligned} \textsf{D}(B_q):= \big \{f=(f_1,f_2)\in W^{2,q}(\mathcal {O};\mathbb {R}^2)\,:\,&f|_{\Gamma _b}=0, \\&f_2 |_{\Gamma _u}=\partial _z f_1|_{\Gamma _u}=0\big \}. \end{aligned}$$

Note that $B_q$ has the same boundary conditions of $A_q$. However, $B_q$ is considerably more simple than $A_q$ since, to study its spectral properties, it is possible to use reflection arguments which are not available for the Stokes operator, cf. Appendix 1.

We prove Theorem 2.1 by using the transference techniques developed in [42]. By [42, Theorem9], we divide the proof into the following steps:

1. Boundedness of the $H^{\infty }$-calculus for $A_2$ and $B_2$ (i.e. in the Hilbertian case).
2. Boundedness of the $H^{\infty }$-calculus for $B_q$ for all $q\in (1,\infty )$.
3. $$\mathcal {R}$$-sectoriality of $$A_q$$.
4. Conclusion via transference and interpolation [42, Theorems 5 and 9].

### A.1 The Hilbertian case

Here we analyse the $L^2$-case of Theorem 2.1, i.e. the operators $A:=A_2$ and $B:=B_2$ acting on $\mathbb {L}^2$ and $l^2(\mathcal {O};\mathbb {R}^2)$, respectively.

#### Proposition A.1

*A* and *B* are positive self-adjoint operators, and

A.1 $$\begin{aligned} \begin{aligned} \textsf{D}(A^{\gamma })=\textsf{D}((A^*)^{\gamma })=\mathbb {H}^{2\gamma }(\mathcal {O}),\quad \textsf{D}(B^{\gamma })=\textsf{D}((B^*)^{\gamma })= H^{2\gamma }(\mathcal {O};\mathbb {R}^2) \end{aligned} \end{aligned}$$

for all $$\gamma \in (0,\tfrac{1}{4})$$.

The above result and [32, Proposition 10.2.23] imply that *A* and *B* have bounded $H^{\infty }$-calculus of angle 0. Below we mainly focus on the operator *A*. The argument to treat *B* is analogous and simpler.

Consider the elliptic problem associated to *A*, i.e.

A.2 $$\begin{aligned} \left\{ \begin{aligned} -\Delta u+\nabla \pi&=f, \qquad&\text { on }&\mathcal {O},\\ \textrm{div}\, u&=0,&\text { on }&\mathcal {O}, \\ u&=0,&\text { on }&\Gamma _b,\\ \partial _{z}u_1&=0,\qquad&\text { on }&\Gamma _u,\\ u_2&=0,\qquad&\text { on }&\Gamma _u. \end{aligned}\right. \end{aligned}$$

If $f\in V^*$, the definition of weak solutions $u\in V$ is standard and similar to the one of (2.1). The well-posedness of (A.2) is analysed below.

#### Proposition A.2

For each $f\in V^*$ there exists a unique solution of problem (A.2).

Moreover if $f\in H$ then $u\in \textsf{D}(A)$ and

$$\begin{aligned} \Vert u\Vert _{\textsf{D}(A)}+\Vert \pi \Vert _{H^1/\mathbb {R}}\le C\Vert f\Vert . \end{aligned}$$

#### Proof

Existence of weak solutions follows immediately by Lax–Milgram Lemma, since Poincaré inequality holds in *V*. Therefore we can endow *V* with the norm $\Vert \nabla u\Vert _V:=\Vert \nabla u\Vert _{L^2}$ equivalent to the standard $H^1$ norm. Let now $f\in H$, therefore the weak formulation satisfied by *u* reduces to

A.3 $$\begin{aligned} \langle \nabla u,\nabla \phi \rangle =\langle f,\phi \rangle \quad \forall \phi \in V . \end{aligned}$$

Considering $\phi \in \mathcal {D}(\mathcal {O})$ it follows that

$$\begin{aligned} \langle \Delta u+f,\phi \rangle _{\mathcal {D}(\mathcal {O})', \mathcal {D}(\mathcal {O})}=0, \end{aligned}$$

therefore by [51, Proposition 1.1, Proposition 1.2] it exists $\pi \in L^2(D)$ such that

A.4 $$\begin{aligned} -\Delta u+\nabla \pi =f \end{aligned}$$

in the sense of distributions and

$$\begin{aligned} \Vert \pi \Vert _{L^2/\mathbb {R}}\le C \Vert \Delta u\Vert _{H^{-1}}+\Vert f\Vert \lesssim \Vert f\Vert . \end{aligned}$$

The higher regularity follows by the standard Niremberg’s method of finite difference quotients. Therefore, fix $h>0$, extending periodically either *u* and *f* in the *x* direction and consider $\phi =\tau _{h}\tau _{-h} u$ as a test function in (A.3), where $\tau _h v=\frac{v(x+h,z)-v(x,z)}{h}.$ Then by change of variables it follows that

$$\begin{aligned} \Vert \tau _{-h}\nabla u\Vert \le C\Vert f\Vert . \end{aligned}$$

The relation above implies that

$$\begin{aligned} \Vert \partial _x\nabla u\Vert \le C\Vert f\Vert . \end{aligned}$$

Let us now consider $\phi =\partial _x\psi ,\ \psi \in \mathcal {D}(\mathcal {O})$ as test function in (A.3). Therefore arguing as above it follows that $\partial _x \pi \in L^2(\mathcal {O})$ and $\Vert \partial _x\pi \Vert _{L^2}\lesssim \Vert f\Vert$. Since Eq. (A.4) is satisfied in the sense of distribution and *u* is divergence free it follows that

$$\begin{aligned} \partial _z \pi =f_2+\partial _{xx}u_2-\partial _{xz}u_1\in L^2(\mathcal {O}). \end{aligned}$$

This implies that $\Vert \nabla \pi \Vert _{L^2}\lesssim \Vert f\Vert .$ Lastly $u_1$ satisfies

$$\begin{aligned} -\partial _{zz}u_1&=-\partial _x\pi +\partial _{xx}u_1+f_1\in L^2(\mathcal {O}), \end{aligned}$$

which completes the proof. $\square$

We are ready to prove Proposition A.1.

#### Proof of Proposition A.1

*Step 1:* *A* *and* *B* *are a positive self-adjoint operators*. As above we only discuss the operator *A*. The positivity of *A* is clear. Next, note that, integrating by parts

$$\begin{aligned} \langle A u,v\rangle =\langle u,A v\rangle \quad \forall u,v\in \textsf{D}(A). \end{aligned}$$

This means that *A* is symmetric. It remains to show that $\textsf{D}(A^*)=\textsf{D}(A)$ and $\forall u\in \textsf{D}(A),\ A^*u=Au$. By definition

$$\begin{aligned} \textsf{D}(A^*)=\{u \in H:\quad&F: D(A)\subseteq H\rightarrow \mathbb R,\quad F(v)=\langle u,Av\rangle \\ {}&\text { has a linear bounded extension on } H \}. \end{aligned}$$

For each $u\in \textsf{D}(A^*),\ F(v)=\langle u,Av\rangle =\langle f_u,v\rangle$ therefore $A^*u=f_u$. In particular, $\forall u\in \textsf{D}(A^*)\ \langle u,Av\rangle =\langle A^*u,v\rangle$. Thanks to the fact that *A* is symmetric we have $\textsf{D}(A)\subseteq \textsf{D}(A^*)$. Given now $v\in \textsf{D}(A^*),\ f_v=A^*v\in H$, let us consider the boundary value problem (A.2) with forcing term equal to $f_v$. By Proposition A.2 it has a unique solution $(w,\pi )\in \textsf{D}(A)\times H^1(\mathcal {O})$, this implies that $Aw=f_v=A^*v$. For each $z\in H$, let us consider the boundary value problem (A.2) with forcing term equal to *z*. By Proposition A.2 it exists a unique $S_z\in \textsf{D}(A)$ such that $As_z=z$. Therefore $\langle z,w-v\rangle =\langle As_z,w-v\rangle =\langle s_z,Aw-A^*v\rangle =0$ thanks to the fact that *A* is symmetric. Since *z* is arbitrary, then $v=w$ and the claim follows.

*Step 2: Proof of *(A.1). We begin by proving the first identity in (A.1). Note that $\textsf{D}(B^{\gamma })=\textsf{D}((B^*)^{\gamma })$ for $\gamma <1/2$ follows from [34, Theorem 1.1] and Step 1. By Step 1 and [32, Proposition 10.2.23], *B* has bounded $H^{\infty }$-calculus and in particular *B* has the bounded imaginary powers property, [43, Subsection 3.4]. By [43, Theorem 3.3.7], $\textsf{D}(B^{\gamma })=[L^2(\mathcal {O};\mathbb {R}^2),\textsf{D}(B)]_{\gamma }$ for all $\gamma <1$. The latter gives $\textsf{D}(B^{\gamma })=H^{2\gamma }(\mathcal {O};\mathbb {R}^2)$ in case $\gamma <1/4$ by [44]. The second identity in (A.1) follows analogously, where one uses the argument in [28] (see also [48, Proposition 5.5, Chapter 17]) to deduce $\textsf{D}(A^{\gamma })=\mathbb {H}^{2\gamma }(\mathcal {O})$ from the first identity in (A.1).

### A.2 Bounded $H^{\infty }$-calculus for Laplace operators

In this subsection we prove the boundedness of the $H^{\infty }$-calculus for $B_q$. The basic idea is to use the product structure of the domain $\mathcal {O}$ and to write $B_{q}u=(L_{q,\textrm{R}}u_1,L_{q,\textrm{D}}u_2)$ where

$$\begin{aligned} \textsf{D}(L_{q,\textrm{D}})&:=\big \{f\in W^{2,q}(\mathcal {O})\,:\, f|_{\Gamma _b}=f|_{\Gamma _u}=0 \big \},{} & {} L_{q,\textrm{D}}f:= \Delta f,\\ \textsf{D}(L_{q,\textrm{R}})&:=\big \{f\in W^{2,q}(\mathcal {O})\,:\, \partial _z f|_{\Gamma _b}=0,\ f|_{\Gamma _u}=0 \big \},{} & {} L_{q,\textrm{R}}f:= \Delta f. \end{aligned}$$

#### Proposition A.3

(Bounded $H^{\infty }$-calculus for Laplace operators) Let $q\in (1,\infty )$ and let $\mathcal {O}$ be as above. Then $-L_{q,\textrm{D}}$ and $-L_{q,\textrm{R}}$ have a bounded $H^{\infty }$-calculus of angle 0. In particular $B_q$ has a bounded $H^{\infty }$-calculus of angle 0.

The above statement also holds for the Neumann Laplacian, but it will not be needed below.

#### Proof

We divide the proof into three steps. In the first step, we exploit the product structure of our domain to reduce the problem to a one dimensional situation.

*Step 1: Reduction to the 1d case*. Then the Dirichlet and the Robin Laplacian in 1d are given by

$$\begin{aligned} \textsf{D}(\ell _{q,\textrm{D}})&:=\big \{f\in W^{2,q}(0,a)\,:\, f(0)=f(a)=0 \big \},{} & {} \ell _{q,\textrm{R}}f:= \partial _x^2 f,\\ \textsf{D}(\ell _{q,\textrm{R}})&:=\big \{f\in W^{2,q}(0,a)\,:\, \partial _x f(0)= f(a)=0 \big \},{} & {} \ell _{q,\textrm{R}}f:= \partial _x^2 f. \end{aligned}$$

Let us consider $\ell _{q,\textrm{D}}$, the other case is analogue. In this step we assume that $-\ell _{q,\textrm{D}}$ has a bounded $H^{\infty }$-calculus of angle 0. Let $\ell _{q,\textrm{P}}$ be the Laplacian on the periodic torus $\mathbb {T}$ with domain $W^{2,q}(\mathbb {T})$. The boundedness of the $H^{\infty }$-calculus for $-\ell _{q,\textrm{P}}$ such operator follows from the periodic version of [32, Theorem 10.2.25] and $\omega _{H^{\infty }}(\ell _{q,\textrm{P}})=0$.

On $L^2(\mathcal {O})$ considers the operator

$$\begin{aligned} (\ell _{q,\textrm{D}}^{(x)}f)(x,z)= (\ell _{q,\textrm{D}}f(\cdot ,z))(x), \qquad (\ell _{q,\textrm{P}}^{(y)}f)(x,z)= (\ell _{q,\textrm{D}}f(x,\cdot ))(z), \end{aligned}$$

with the corresponding natural domains. It is clear that both $-\ell _{q,\textrm{D}}^{(x)}$ and $-\ell _{q,\textrm{P}}^{(z)}$ have bounded $H^{\infty }$-calculus of angle 0. Now by sum of commuting operators [43, Corollary 4.5.8], the sum operator $-A_q:=-\ell _{q,\textrm{D}}^{(x)}-\ell _{q,\textrm{P}}^{(z)}$ has a bounded $H^{\infty }$-calculus of angle 0 with domain

$$\begin{aligned} \textsf{D}(A_q)=\textsf{D}(\ell _{q,\textrm{D}}^{(x)}) \cap \textsf{D}(\ell _{q,\textrm{P}}^{(z)})= \textsf{D}(L_{q,\textrm{D}}) \end{aligned}$$

where the last equality follows from elliptic regularity.

*Step 2:* $-L_{q,\textrm{D}}$ *has a bounded* $H^{\infty }$-*calculus of angle* 0. By rescaling and translation we may replace (0, *a*) by $(-\pi ,\pi )$. Let $\ell _{q,\textrm{P}}$ be the Laplacian on the periodic torus $\mathbb {T}=(-\pi ,\pi )$ (as measure space) with domain $W^{2,q}(\mathbb {T})$. Let

$$\begin{aligned} Y:=\big \{f\in L^2(\mathbb {T}):\, f=\sum _{n\ge 0} f_n \sin (n x) \text { where } (f_n)_{n\ge 1} \in \ell ^2 \big \}. \end{aligned}$$

It is clear that $Y\subseteq L^2(\mathbb {T})$ is closed, and

$$\begin{aligned} (\lambda - \ell _{q,\textrm{P}})^{-1}: Y\rightarrow Y\ \ \text { for all }\ \ \lambda \in \rho (L_{q,\textrm{D}}). \end{aligned}$$

Now note that $L_{q,\textrm{D}}$ is the part of $\ell _{q,\textrm{P}}$ on *Y*, i.e.

$$\begin{aligned} \textsf{D}(L_{q,\textrm{D}})&=\{f\in \textsf{D}(\ell _{q,\textrm{P}}) \cap Y\,:\, \ell _{q,\textrm{P}}f\in Y\}, \\ L_{q,\textrm{D}}f&= \ell _{q,\textrm{P}}f \ \ \text { for all }f \in \textsf{D}(L_{q,\textrm{D}}). \end{aligned}$$

Now the claim of Step 1 follows from [32, Proposition10.2.18] and the periodic version of [32, Theorem10.2.25].

*Step 2:* $-L_{q,\textrm{R}}$ *has a bounded* $H^{\infty }$-*calculus of angle* 0. As in the above step, by rescaling we replace (0, *a*) by $(0,\pi )$. Consider the reflection map

$$\begin{aligned} Rf(z):= {\left\{ \begin{array}{ll} f(z) \quad &{}z\in (0,\pi ),\\ f(-z)\quad &{}z\in (-\pi ,0). \end{array}\right. } \end{aligned}$$

Let $L_{q,\textrm{D}}$ be the Dirichlet Laplacian on $(-\pi ,\pi )$. Then one can readily check that $\rho (L_{q,\textrm{D}})\subseteq \rho (L_{q,\textrm{R}})$ and for all $\lambda \in \rho (L_{q,\textrm{D}})$

$$\begin{aligned} (\lambda -L_{q,\textrm{R}})^{-1} f =\big [(\lambda - L_{q,\textrm{D}})^{-1} Rf \big ] \Big |_{(0,\pi )}. \end{aligned}$$

Now the claim follows from Step 1 and the definition of $H^{\infty }$-calculus. $\square$

### A.3 $\mathcal {R}$-sectoriality for the Stokes operator

For the notion of $\mathcal {R}$-boundedness of a family of linear operators we refer to [32, Chapter 8]. For a family of linear operators $\mathscr {J}$, the $\mathcal {R}$-bound is denoted by $\mathcal {R}(\mathscr {J})$. As in [32, Chapter 10], we said that operator *T* on a Banach space *X* is said to be $\mathcal {R}$-sectorial if there exists $\phi \in (0,\pi )$ such that $\rho (A)\subseteq \{\lambda \in \mathbb {C}\,|\, |\arg \lambda |\ge \pi -\phi \}$ and

$$\begin{aligned} \mathcal {R}(\lambda (\lambda -T)^{-1}\,|\, |\arg \lambda |>\pi -\phi )<\infty . \end{aligned}$$

The $\mathcal {R}$-sectoriality angle is the infimum over all $\phi \in (0,\pi )$ for which the above holds. The main result of this subsection reads as follows.

#### Proposition A.4

*For all* $q\in (1,\infty )$, *the operator* $A_q$ *is* $\mathcal {R}$-*sectorial with* $\mathcal {R}$-*sectoriality angle* $<\pi /2$.

#### Proof

Fix $q\in (1,\infty )$. For simplicity we first prove the statement for a shifted Stokes operator and in a second step we conclude by a simple translation argument.

*Step 1: There exists* $\lambda _q$ *such that* $\lambda _q+A_q$*is* $\mathcal {R}$-*sectorial with* $\mathcal {R}$-*sectoriality angle* $<\pi /2$. Due to the the well-known equivalence of maximal $L^q$-regularity and $\mathcal {R}$-sectoriality proven by L. Weis [54] (see also [43, Subsection 4.2, Chapter 3]), it is enough to show that, for all $f\in L^q(0,1;\mathbb {L}^q)$, the Stokes problem on $\mathcal {O}$,

A.5 $$\begin{aligned} \left\{ \begin{aligned} \partial _t u&=\Delta u-\nabla P +f,&\ \ \ \text { on }&(0,1)\times \mathcal {O},\\ \textrm{div}\, u&=0,&\text { on }&(0,1)\times \mathcal {O}, \\ u&=0,&\text { on }&(0,1)\times \Gamma _b, \\ u_2&= \partial _z u_1=0,&\text { on }&(0,1)\times \Gamma _u,\\ u(0)&=0,&\text { on }&\mathcal {O}, \end{aligned} \right. \end{aligned}$$

admits a unique solution in the class

A.6 $$\begin{aligned} u\in W^{1,q}(0,1;\mathbb {L}^q)\cap L^q(0,1;\mathbb {W}^{2,q}(\mathcal {O})), \quad P\in L^q(0,1;W^{1,q}(\mathcal {O})). \end{aligned}$$

The proof follows a standard localization argument. Let $(\phi _j)_{j=1}^N$ be a smooth partition of the unity such that, for all $j\in \{1,\dots ,N\}$, $\textrm{diam}(\textrm{supp}\,\phi _k)<\frac{1}{2}$

$$\begin{aligned} \text { either }\quad \textrm{supp}\,\phi _j \cap (\mathbb {T}\times \{0\})=\varnothing \quad \text { or }\quad \textrm{supp}\,\phi _j\cap (\mathbb {T}\times \{a\})=\varnothing . \end{aligned}$$

Fix $k\in \{1,\dots ,N\}$. Multiplying (A.5) by $\phi _k$, we obtain for $u_{k}:=\phi _k u$ and $P_k=\phi _k P$ either

A.7 $$\begin{aligned} \left\{ \begin{aligned} \partial _t u_k&=\Delta u_k-\nabla P_k +\phi _k f + \mathcal {L}_k (u,P),&\text { on }&(0,1)\times \mathbb {R}\times (0,\infty ),\\ \textrm{div}\, u_k&=\nabla \phi _k \cdot u,&\text { on }&(0,1)\times \mathbb {R}\times (0,\infty ), \\ u_k&=0,&\text { on }&(0,1)\times \mathbb {R}\times \{0\}, \\ u_k(0)&=0,&\text { on }&\mathbb {R}\times (0,\infty ), \end{aligned} \right. \end{aligned}$$

or

A.8 $$\begin{aligned} \left\{ \begin{aligned} \partial _t u_k&=\Delta u_k-\nabla P_k +\phi _k f+ \mathcal {L}_k (u,P),&\text { on }&(0,1)\times \mathbb {R}\times (-\infty ,a),\\ \textrm{div}\, u_k&=\nabla \phi _k \cdot u,&\text { on }&(0,1)\times \mathbb {R}\times (-\infty ,a),\\ \phi _k u_2&=0,&\text { on }&(0,1)\times \mathbb {R}\times \{a\},\\ \partial _z (\phi _k u_1)&= u_1 \partial _z \phi _k,&\text { on }&(0,1)\times \mathbb {R}\times \{a\},\\ u_k(0)&=0,&\text { on }&\mathbb {R}\times (-\infty ,a). \end{aligned} \right. \end{aligned}$$

Here $\mathcal {L}_k$ denotes a lower order operator w.r.t. to the maximal regularity space for (*u*, *P*) in (A.6).

Maximal $L^p(L^q)$-regularity estimates for (A.7) and (A.8) are proven in [43, Theorem 7.2.1] in the case of no-slip or pure-slip, respectively (see conditions (7.16) and (7.17) on [43, p. 323]). Now a-priori estimates for solutions as in (A.6) in the maximal $L^p(L^q)$-regularity class $W^{1,q}(0,1;\mathbb {L}^q)\cap L^q(0,1;\mathbb {W}^{2,q}(\mathcal {O}))$ follows by repeating the localization argument of [43, Subsection 3.4 in Chapter 7] to adsorb the lower order terms.

It remains to discuss the existence of solutions as in (A.6). Arguing as in step 3 of Theorem 2.2 and using $L^2$-theory, one can prove existence of smooth solutions to equation (A.5) in case of smooth data *f*. Hence the existence follows from a standard density argument and the a-priori estimates obtained above for solutions in the class $W^{1,q}(0,1;\mathbb {L}^q)\cap L^q(0,1;\mathbb {W}^{2,q}(\mathcal {O}))$.

*Step 2: Conclusion*. By Step 1, it remains to remove the shift $\lambda _q$. Arguing as in [1, Proposition 2.2], by holomorphicity of the resolvent and [43, Proposition 4.1.12], it is enough to show that

$$\begin{aligned} \rho (A_q)\subseteq \{\lambda \in \mathbb {C}\,|\, |\arg z|>\psi \}, \quad \text { for some }\psi <\pi /2. \end{aligned}$$

In the case $q>2$, noticing that $(\lambda - A_q) = (\lambda -\lambda _q-A_q) +\lambda _q$ and that $\rho (\lambda _q+A_q)\subseteq \{\lambda \in \mathbb {C}\,|\, |\arg z|>\phi \}$ for some $\phi <\pi /2$ by Step 1, the conclusion can be obtained by using a standard bootstrap method via Sobolev embeddings.

In the case $q<2$ one uses $(A_q)^*=A_{q'}$. $\square$

### A.4 Proof of Theorem 2.1

As the proof of Theorem 2.1 follows the one of [42, Theorem 16], we only provide a sketch.

#### Proof of Theorem 2.1—Sketch

*Step 1: There exists* $\beta >0$ *for which the following estimates hold*:

A.9 $$\begin{aligned} \begin{aligned} \sup _{1\le t,s\le 2} \mathcal {R}(\varphi (2^{j+\ell } s A_2) \psi (t2^{j} B_2))&\lesssim 2^{-\beta \ell },\\ \sup _{1\le t,s\le 2} \mathcal {R}(\varphi (2^{j+\ell }s A_2)^*\mathbb {P}_{p}^*\psi (t2^{j} B_2)^* )&\lesssim 2^{-\beta \ell }. \end{aligned} \end{aligned}$$

Recall that $\mathcal {R}(\mathscr {J})$ stand for the $\mathcal {R}$-bound of the family of operators $\mathscr {J}$, see [32, Chapter 9] for details on $\mathcal {R}$-boundedness.

By elliptic regularity we have $\mathbb {P}: H^{1}(\mathcal {O};\mathbb {R}^2)\rightarrow \mathbb {H}^{1}(\mathcal {O})$. Interpolating we obtain $\mathbb {P}: H^{s}(\mathcal {O};\mathbb {R}^2)\rightarrow \mathbb {H}^{s}(\mathcal {O};\mathbb {R}^2)$ for all $s\in (0,1)$. Hence $\mathbb {P}: \textsf{D}(B^{\gamma })\rightarrow \textsf{D}(A^{\gamma })$ for all $\gamma \in (0,1/4)$. The estimate (A.9) now follows from [42, Proposition 10] and (A.1).

*Step 2: Boundedness of the* $H^{\infty }$-*calculus*. Next we argue as in the proof of [42, Theorem 5]. Let $q\in (1,\infty )$ be as in the statement of Theorem 2.1 and fix $p\in (q,\infty )$. By $\mathcal {R}$-sectoriality of $A_p$ and $B_p$ (i.e. Proposition A.4) and [32, Proposition 10.3.2],

A.10 $$\begin{aligned} \mathcal {R}(\varphi (t A_p):\, t>0 )\le c_0 \quad \text { and }\quad \mathcal {R}(\psi (s B_p):\, s>0)\le c_0. \end{aligned}$$

Note that $(A_r)_{r\in (1,\infty )}$, $(B_r)_{r\in (1,\infty )}$ are consistent family of operators. Hence, by complex interpolation and [32, Proposition 8.4.4], we have that (A.9) holds for some $\beta =\beta (r,p)>0$ and with $(A_2,B_2)$ replaced by $(A_q,B_q)$. Now the boundedness of the $H^{\infty }$-calculus follows from [42, Theorem 9].

*Step 3: Description of the fractional powers*. To obtain the description of the fractional powers of $A_q$ and $B_q$ one can argue as in the proof of (A.1) by using the bounded imaginary power property and [28, 44].

## References

[1] Agresti, A., Hussein, A.: Maximal -regularity and -calculus for block operator matrices and applications. J. Funct. Anal. **285**(11), 110146 (2023)

[2] Agresti, A., Veraar, M.: Stability properties of stochastic maximal -regularity. J. Math. Anal. Appl. **482**(2), 123553 (2020)

[3] Agresti, A., Lindemulder, N., Veraar, M.: On the trace embedding and its applications to evolution equations. Math. Nachr. **296**(4), 1319–1350 (2023)

[4] Alòs, E., Bonaccorsi, S.: Stochastic partial differential equations with Dirichlet white-noise boundary conditions. Ann. l’IHP Probab. Stat. **38**(2), 125–154 (2002)

[5] Amann, H.: Linear and Quasilinear Parabolic Problems, vol. 1. Springer, New York (1995)

[6] Ambrosio, L., Carlotto, A., Massaccesi, A.: Lectures on Elliptic Partial Differential Equations, vol. 18. Springer, New York (2019)

[7] Angenent, S.: Nonlinear analytic semiflows. Proc. R. Soc. Edinb. Sect. A **115**(1–2), 91–107 (1990)

[8] Angenent, S.: Parabolic equations for curves on surfaces. I. Curves with -integrable curvature. Ann. Math. (2) **132**(3), 451–483 (1990)

[9] Antonio Agresti and Mark Veraar: Nonlinear parabolic stochastic evolution equations in critical spaces part I. Stochastic maximal regularity and local existence. Nonlinearity **35**(8), 4100 (2022)

[10] Bergh, J., Löfström, J.: Interpolation Spaces: An Introduction, vol. 223. Springer, New York (2012)

[11] Berselli, L.C., Romito, M.: On the existence and uniqueness of weak solutions for a vorticity seeding model. SIAM J. Math. Anal. **37**(6), 1780–1799 (2006)

[12] Bessaih, H., Maris, F.: Homogenization of the stochastic Navier–Stokes equation with a stochastic slip boundary condition. Appl. Anal. **95**(12), 2703–2735 (2016)

[13] Bessaih, H., Efendiev, Y., Maris, F.: Homogenization of the evolution Stokes equation in a perforated domain with a stochastic Fourier boundary condition. Netw. Heterog. Media **10**(2), 343–367 (2015)

[14] Binz, T., Hieber, M., Hussein, A. & Saal, M. The primitive equations with stochastic wind driven boundary conditions. arXiv preprint arXiv:2009.09449 (2020)

[15] Bonaccorsi, S., Zanella, M.: Absolute continuity of the law for solutions of stochastic differential equations with boundary noise. Stoch. Dyn. **17**(6), 1750045 (2017)

[16] Brzeźniak, Z., Goldys, B., Peszat, S., Russo, F.: Second order PDEs with Dirichlet white noise boundary conditions. J. Evolut. Equ. **15**(1), 1–26 (2015)

[17] Da Prato, G., Debussche, A.: Two-dimensional Navier–Stokes equations driven by a space-time white noise. J. Funct. Anal. **196**(1), 180–210 (2002)

[18] Da Prato, G., Zabczyk, J.: Evolution equations with white-noise boundary conditions. Stoch. Int. J. Probab. Stoch. Process. **42**(3–4), 167–182 (1993)

[19] Da Prato, G., Zabczyk, J.: Ergodicity for Infinite Dimensional Systems, vol. 229. Cambridge University Press, Cambridge (1996)

[20] Da Prato, G., Zabczyk, J.: Stochastic Equations in Infinite Dimensions. Cambridge University Press, Cambridge (2014)

[21] Dalibard, A.-L.: Asymptotic behavior of a rapidly rotating fluid with random stationary surface stress. SIAM J. Math. Anal. **41**(2), 511–563 (2009)

[22] Dalibard, A.-L., Saint-Raymond, L.: Mathematical study of rotating fluids with resonant surface stress. J. Differ. Equ. **246**(6), 2304–2354 (2009)

[23] Debussche, A., Fuhrman, M., Tessitore, G.: Optimal control of a stochastic heat equation with boundary-noise and boundary-control. ESAIM Control Optim. Calculus Var. **13**(1), 178–205 (2007)

[24] Desjardins, B., Grenier, E.: On the homogeneous model of wind-driven ocean circulation. SIAM J. Appl. Math. **60**(1), 43–60 (1999)

[25] Fabbri, G., Goldys, B.: An LQ problem for the heat equation on the halfline with Dirichlet boundary control and noise. SIAM J. Control Optim. **48**(3), 1473–1488 (2009)

[26] Flandoli, F., Luongo, E.: Heat diffusion in a channel under white noise modeling of turbulence. Math. Eng. **4**(4), 1–21 (2022)

[27] Flandoli, F., Luongo, E.: Stochastic partial differential equations in fluid mechanics. Lecture Notes in Mathematics, vol. 2330. Springer, Singapore (2023)

[28] Fujita, H., Morimoto, H.: On fractional powers of the Stokes operator. Proc. Jpn. Acad. **46**(10), 1141–1143 (1970)

[29] Galdi, G.: An Introduction to the Mathematical Theory of the Navier–Stokes Equations: Steady-State Problems. Springer, New York (2011)

[30] Gill, A.E.: Atmosphere–Ocean Dynamics, vol. 30. Academic press, New York (1982)

[31] Goldys, B., Peszat, S.: Linear parabolic equation with Dirichlet white noise boundary conditions. J. Differ. Equ. **362**, 382–437 (2023)

[32] Hytönen, T., van Neerven, J., Veraar, M., Weis, L.: Analysis in Banach spaces. Vol. II. Probabilistic Methods and Operator Theory. In: Ergebnisse der Mathematik und ihrer Grenzgebiete 3. Folge, vol. 67. Springer, New York (2017)

[33] Ju, N.: On -solutions and -weak solutions of the 3D primitive equations. Indiana Univ. Math. J. **3**, 973–996 (2017)

[34] Kato, T.: Fractional powers of dissipative operators. J. Math. Soc. Jpn. **13**(3), 246–274 (1961)

[35] Kozono, H., Yanagisawa, T.: Generalized Lax–Milgram theorem in Banach spaces and its application to the elliptic system of boundary value problems. Manuscr. Math. **141**(3–4), 637–662 (2013)

[36] Lemarié-Rieusset, P.G.: The Navier–Stokes Problem in the 21st Century. CRC Press, New York (2018)

[37] Lions, P.-L.: Mathematical Topics in Fluid Mechanics: Incompressible Models, vol. 1. Clarendon Press, Oxford (1996)

[38] Lions, J.-L., Temam, R., Wang, S.H.: Models for the coupled atmosphere and ocean, (CaO I, II). Comput. Mech. Adv. **1**, 3–119 (1993)

[39] Lorist, E., Veraar, M.: Singular stochastic integral operators. Anal. PDE **14**(5), 1443–1507 (2021)

[40] Pedlosky, J.: Ocean Circulation Theory. Springer, New York (1996)

[41] Pedlosky, J.: Geophysical Fluid Dynamics. Springer, New York (2013)

[42] Peer Christian Kunstmann and Lutz Weis: New criteria for the -calculus and the Stokes operator on bounded Lipschitz domains. J. Evol. Equ. **17**(1), 387–409 (2017)

[43] Prüss, J., Simonett, G.: Moving interfaces and quasilinear parabolic evolution equations. In: Monographs in Mathematics, vol. 105. Birkhäuser/Springer, New York (2016)

[44] Robert Thomas Seeley: Interpolation in with boundary conditions. Stud. Math. **44**(1), 47–60 (1972)

[45] Sawano, Y.: Theory of Besov spaces. In: Developments in Mathematics, vol. 56. Springer, Singapore (2018)

[46] Schmeisser, H.-J., Triebel, H.: Topics in Fourier Analysis and Function Spaces. Wiley, New York (1987)

[47] Serrin, J.: On the Interior Regularity of Weak Solutions of the Navier–Stokes Equations. Mathematics Division/Air Force Office of Scientific Research, Arlington (1961)

[48] Taylor, M.E.: Partial differential equations III. Nonlinear equations. In: Applied Mathematical Sciences, vol. 117, 2nd edn. Springer, New York (2011)

[49] Taylor, M.E. Partial differential equations I. Basic theory. In: Applied Mathematical Sciences, vol. 115, 2nd edn. Springer, New York (2011)

[50] Temam, R.: Navier–Stokes Equations and Nonlinear Functional Analysis. SIAM, Philadelphia (1995)

[51] Temam, R.: Navier–Stokes Equations: Theory and Numerical Analysis, vol. 343. American Mathematical Society, New York (2001)

[52] van Neerven, J., Veraar, M., Weis, L.: Stochastic integration in Banach spaces—a survey. In: Stochastic Analysis: A Series of Lectures: Centre Interfacultaire Bernoulli, January–June 2012, Ecole Polytechnique Fédérale de Lausanne, Switzerland, pp. 297–332. Springer, New York (2015)

[53] van Neerven, J., Veraar, M., Weis, L.: Stochastic maximal -regularity. Ann. Probab. **40**(2), 788–812 (2012)

[54] Weis, L.: Operator-valued Fourier multiplier theorems and maximal -regularity. Math. Ann. **319**(4), 735–758 (2001)

